# Altered hepatic metabolic landscape and insulin sensitivity in response to pulmonary tuberculosis

**DOI:** 10.1371/journal.ppat.1012565

**Published:** 2024-09-27

**Authors:** Mrinal K. Das, Ben Savidge, John E. Pearl, Thomas Yates, Gareth Miles, Manish Pareek, Pranabashis Haldar, Andrea M. Cooper

**Affiliations:** 1 Department of Respiratory Sciences, Leicester TB Research Group, University of Leicester, Leicester, United Kingdom; 2 Diabetes Research Centre, University of Leicester, Leicester, United Kingdom; 3 NIHR Leicester Biomedical Research Centre, University of Leicester and University Hospitals of Leicester NHS Trust, Leicester, United Kingdom; 4 Leicester Cancer Research Centre, University of Leicester, Clinical Sciences Building, Leicester, United Kingdom; 5 Department of Infection and HIV Medicine, University Hospitals of Leicester NHS Trust, Leicester, United Kingdom; 6 NIHR Respiratory Biomedical Research Centre, Leicester, Glenfield Hospital, Groby Road, Leicester, United Kingdom; Portland VA Medical Center, Oregon Health and Science University, UNITED STATES OF AMERICA

## Abstract

Chronic inflammation triggers development of metabolic disease, and pulmonary tuberculosis (TB) generates chronic systemic inflammation. Whether TB induced-inflammation impacts metabolic organs and leads to metabolic disorder is ill defined. The liver is the master regulator of metabolism and to determine the impact of pulmonary TB on this organ we undertook an unbiased mRNA and protein analyses of the liver in mice with TB and reanalysed published data on human disease. Pulmonary TB led to upregulation of genes in the liver related to immune signalling and downregulation of genes encoding metabolic processes. In liver, IFN signalling pathway genes were upregulated and this was reflected in increased biochemical evidence of IFN signalling, including nuclear location of phosphorylated Stat-1 in hepatocytes. The liver also exhibited reduced expression of genes encoding the gluconeogenesis rate-limiting enzymes Pck1 and G6pc. Phosphorylation of CREB, a transcription factor controlling gluconeogenesis was drastically reduced in the livers of mice with pulmonary TB as was phosphorylation of other glucose metabolism-related kinases, including GSK3a, AMPK, and p42. In support of the upregulated IFN signalling being linked to the downregulated metabolic functions in the liver, we found suppression of gluconeogenic gene expression and reduced CREB phosphorylation in hepatocyte cell lines treated with interferons. The impact of reduced gluconeogenic gene expression in the liver was seen when infected mice were less able to convert pyruvate, a gluconeogenesis substrate, to the same extent as uninfected mice. Infected mice also showed evidence of reduced systemic and hepatic insulin sensitivity. Similarly, in humans with TB, we found that changes in a metabolite-based signature of insulin resistance correlates temporally with successful treatment of active TB and with progression to active TB following exposure. These data support the hypothesis that TB drives interferon-mediated alteration of hepatic metabolism resulting in reduced gluconeogenesis and drives systemic reduction of insulin sensitivity.

## Author summary

Tuberculosis (TB) is a worldwide disease with a substantial proportion of undiagnosed cases. Control of TB is mediated by a protective cellular immune response which is also chronic, inflammatory and systemic. Chronic inflammation can disrupt metabolism and compromise the organs key to maintaining health–such as the liver. The extent to which pulmonary TB can disrupt the metabolic function of the liver is ill defined. We have used a mouse model of pulmonary TB to examine the changes occurring in the liver when immunity is first being expressed in the lung. We found that pulmonary TB leads to upregulation of immune-related signalling pathways in liver cells and that TB infected mice have altered glucose metabolism. Reanalysing published metabolic data from humans, we find that a metabolic signature associated with insulin resistance separates those with TB from healthy subjects and alters with time to progression and resolution of TB, thereby indicating glucose metabolism disruption occurs during TB in humans. These data are important because we show that TB disrupts liver glucose metabolism, and this is critical to understanding the interplay between TB and metabolic disease.

## Introduction

Every year, more than a million patients with tuberculosis (TB) die despite improvements in clinical management, new drugs, and better understanding of the disease [1]. While TB is predominantly a pulmonary disease, symptoms reflect systemic inflammation and disordered metabolism. Patients with severe symptoms, such as loss of muscle mass and fatigue, suffer treatment failure and death and there is an increase in disability adjusted life years on the population level for areas where TB is endemic [2,3]. In addition, the pervasiveness of subclinical TB has now been re-appreciated and the potential burden of TB induced inflammation increased [4]. To reduce the individual and population level impact of both systemic and under diagnosed symptoms, it is important to determine the relative roles of the pathogen and immune response in driving disease. In this regard, the interactive network of type I and type II interferons (IFNs) is thought to be important with type I IFNs driving poor outcomes and type II IFN being essential to control of bacterial growth [5–8].

Metabolomic studies in TB patients and Mtb-infected animal models suggest there is significant dysregulation of systemic metabolism during TB [9–12]. Similarly, metabolic disorders such as diabetes are known risk factors for development and worsening of TB [13,14]. While the lung is the primary organ where Mtb infection and TB disease are seen, the liver is the master-regulator of systemic metabolism and integrates both immunologic and metabolic cues from throughout the body. The liver responds to pathological insults systemically with the release of long-range mediators and intrinsically to immune-mediated signals resulting in altered hepatocyte behaviour [15]. While immune responses and anti-bacterial defence against TB occur in the lung, the liver prepares the body to undergo the stress associated with infection and provides the resource for the metabolically demanding immune response. To ensure that we can intervene to reduce the impact of TB, and to define the ability of TB to contribute as either a driver or indicator of susceptibility to future metabolic disease, the balance between the immune response controlling the bacterium and the changes in metabolism required for an effective immune response must be determined.

In this study we used retrospective analysis of human data sets and an aerosol infection animal model of TB to determine how pulmonary TB and Mtb infection changes metabolic function in the liver. Unbiased ‘Omics analysis supported further investigation into changes in glucose metabolism and insulin signalling both systemically and intrinsically in the liver. Metabolomic analyses of individuals curing from or progressing to TB, indicate a relationship between TB disease and a metabolic surrogate of insulin resistance. Our study shows that metabolic dysregulation occurs concurrently with immune activation during Mtb infection in both mice and humans.

## Results

### TB and Mtb-infection induce immune-metabolic changes in the hepatic transcriptome and proteome

**Fig 1:**
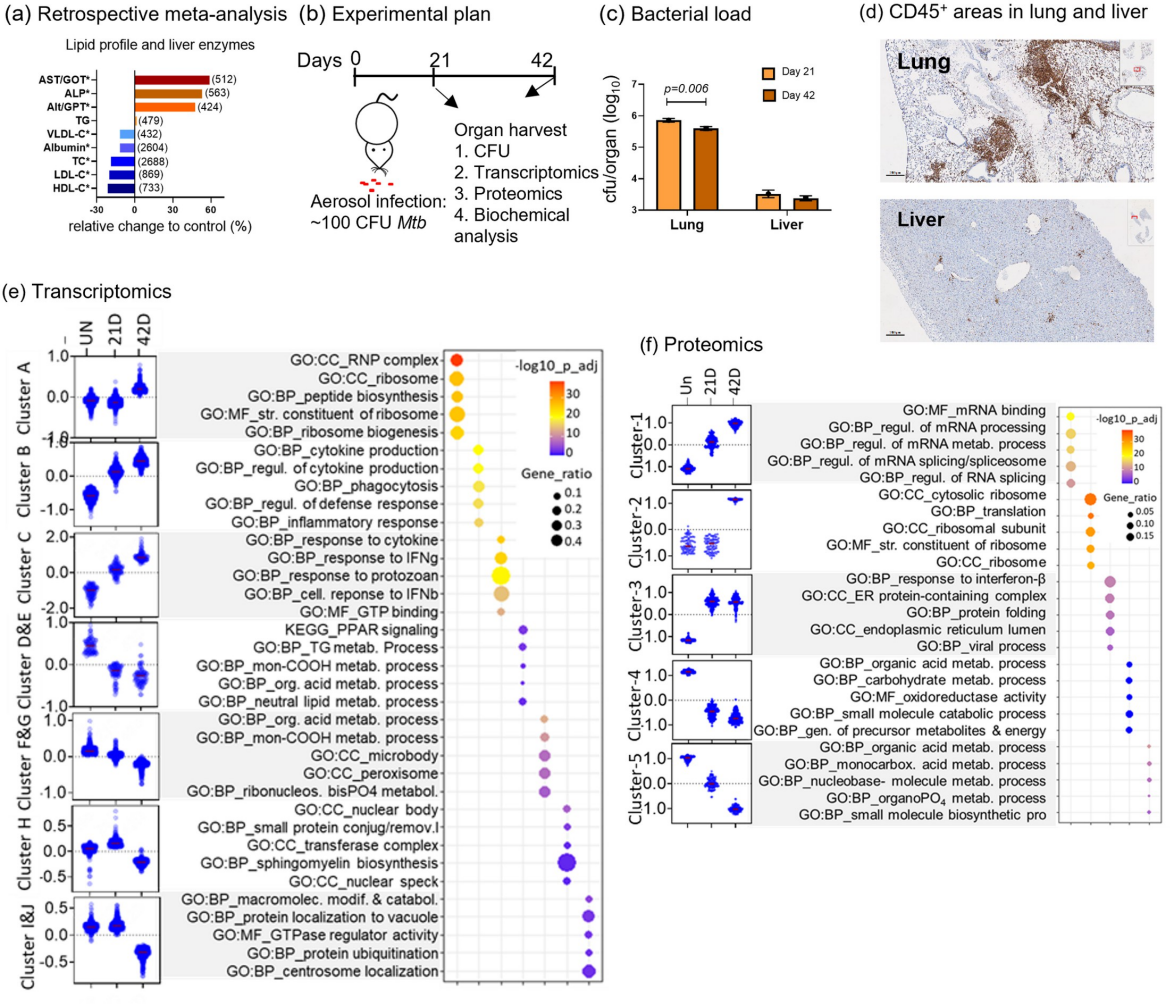
*Mycobacterium tuberculosis* alters the immunometabolic landscape of the liver. (a) Changes in lipid and liver enzyme profiles of adult, drug-naïve TB patients compared to healthy controls, derived from biochemical parameters reported in 15 published articles (details in S1 Data). Number of subjects per parameter are reported in parentheses next to the bar. (b) Experimental design to show days of organ harvest and analysis post aerosol Mtb infection of mice. (c) Bacterial load in lung and liver post infection (n = 10). (d) Immunohistochemical staining of CD45 expression in the lung and liver of 42 day infected mice. (e) K-means cluster analysis of significantly differentially expressed genes (*p*_*ad*j_ <0.05) with the top 5 non redundant pathways listed beside the corresponding clusters (n = 4 mice/group). (f) Cluster analysis of significantly differentially expressed proteins (*FDR* <0.1) by proteomics analysis with the top 5 non redundant pathways listed beside the corresponding clusters (n = 3 mice/group). For (e)-(f) male mice were used.

To define the extent to which liver function is disrupted in adult, drug naïve, TB patients, we undertook a secondary analysis of 15 research articles (details in S1 Data) to determine how far the biochemical parameters of liver health deviate from the healthy baseline in the blood of adult TB patients prior to treatment. We found lower levels of cholesterols and albumin and increased circulation of hepatic transaminases in patients with TB when compared to healthy controls (Fig 1a). Although the elevated hepatic enzyme levels are not altered to a degree that reaches any clinical threshold, these liver specific parameters suggest that there is an impact of pulmonary TB on liver function in a broad survey of human TB patients.

To understand how a localized lung infection may alter liver metabolism, we infected C57BL/6 mice via the aerosol route [16] and determined the impact of the infection on the liver at 21 days, (i.e. when immunity is first expressed in the lung [17] and activated antigen specific T cells are detected in peripheral organs [18] and 42 days (i.e. when immunity has been expressed for 21 days and inflammatory consequences are clearly detectable in tissue) post aerosol (Fig 1b). The bacterial burden was determined in the lung, the primary organ of infection, and in the liver, the primary metabolic organ. As expected from previous work [17–19]), the lung infection remains stable at around one million bacteria per organ, while dissemination to the liver (which begins between day 12–15) results in approximately 3,500 bacteria per organ by day 21 and remains at this level through day 42 (Fig 1c). Histological staining shows extensive accumulation of immune cells as detected by expression of the CD45 marker in the lung, but relatively limited accumulation of these cells in the liver (Fig 1d). Flow cytometric analysis of the cells in the liver did not show extensive fluctuation in the frequencies of CD45 expressing myeloid or lymphoid cells, however there was an increased frequency of activated CD4 and CD8 T cells within the lymphoid compartment in the infected livers (S1B Fig). Importantly mice remained healthy and gained weight throughout the infection (S1 Table). These data show that although the lung is infected and inflamed, the liver undergoes a contained and limited infection and inflammation, and the mice remain healthy by externally observed signs.

To determine the impact of aerosol Mtb infection on liver gene expression over time, intact liver tissue from uninfected and infected mice was rapidly excised, RNA extracted and RNAseq analysis undertaken. We observed infection-induced time course changes in gene expression (S2A Fig). A total of 801 genes were found to be altered (FDR = 0.05, FC = 1.5), (S2 Data) between uninfected (UN) day 21 (21D) and day 42 (42D) post challenge. Major alterations were observed between the uninfected and day 42 samples with 520 upregulated and 163 downregulated genes (S2B Fig). To simplify these patterns, we undertook K means clustering of 2215 significant genes (FDR< 0.05 between any 2 groups, irrespective of fold changes) and this resulted in 10 prominent clusters (genes in each cluster shown in Sheet B in S2 Data). Pathway enrichment analysis of these clusters revealed that upregulated clusters A, B, and C primarily represent immune and translational machinery pathways, while the downregulated clusters D-J represent metabolic functions (Fig 1e). To investigate whether gene expression changes were contributed by the primary cells of the organ or by infiltrating cells from other tissues, we carried out tissue enrichment analysis using the upregulated and downregulated genes as 2 different groups. We found that down regulated genes representing metabolic pathways were liver specific genes whilst upregulated genes representing immune pathways are immune organ specific genes suggesting that immune pathways represent infiltrating cells and activation of resident immune cells, while the down regulated metabolic pathways are occurring in the hepatocytes (S2C Fig).

To determine how changes in the transcriptome impact translational activity, we undertook quantitative proteomics analysis using a TMT-based labelling method of liver tissues from uninfected (Un), day 21 (21D), and day 42 (42D) infected mice. We found that protein expression levels changed as the infection progressed (S2D and S2E Fig). Cluster analysis of the 1215 protein isoforms that were significantly altered (FDR< 0.1 irrespective of fold change) revealed upregulation of proteins involved in translation and transcriptomic machinery along with immune signalling pathways suggesting active biomolecule synthesis in response to infection. Pathways enriched in proteins that were downregulated in response to infection include nucleotide metabolism, carboxylic acid metabolism and carbohydrate metabolism (Fig 1f). Correlation analysis of gene and protein expression relative to uninfected control (fold change) shows a positive correlation between the two ‘Omics data sets (S2F Fig) to a degree reflecting the constraints of transcriptomic/proteomic sampling of complex tissue [20,21]. Overall, these data support the hypothesis that while dissemination of Mtb to the liver and associated inflammation is modest and locally contained, immune activation is increased, and lipid and carbohydrate metabolism is reduced in the liver (specific pathways and associated genes listed in Sheets F and G in S2 Data, and Sheets F and G in S3 Data). Sterile inflammation such as alcoholic hepatitis and diet-induced fibrosis leads to loss of the hepatic identity gene network indicating potential compromised hepatocyte function [22,23]. We undertook a similar gene and protein level network-based approach [23,24] and found that pulmonary Mtb infection also leads to a loss of hepatocyte molecular identity (S2G and S2H Fig) despite modest levels of visible inflammation. These data indicate that modest infection in the liver, alongside substantial infection and inflammation in the lung, can compromise liver function and that the hepatic stress shown by the biochemical markers in TB patients (Fig 1a) may be infection related.

### Pulmonary infection alters gene expression in a tissue specific manner

**Fig 2:**
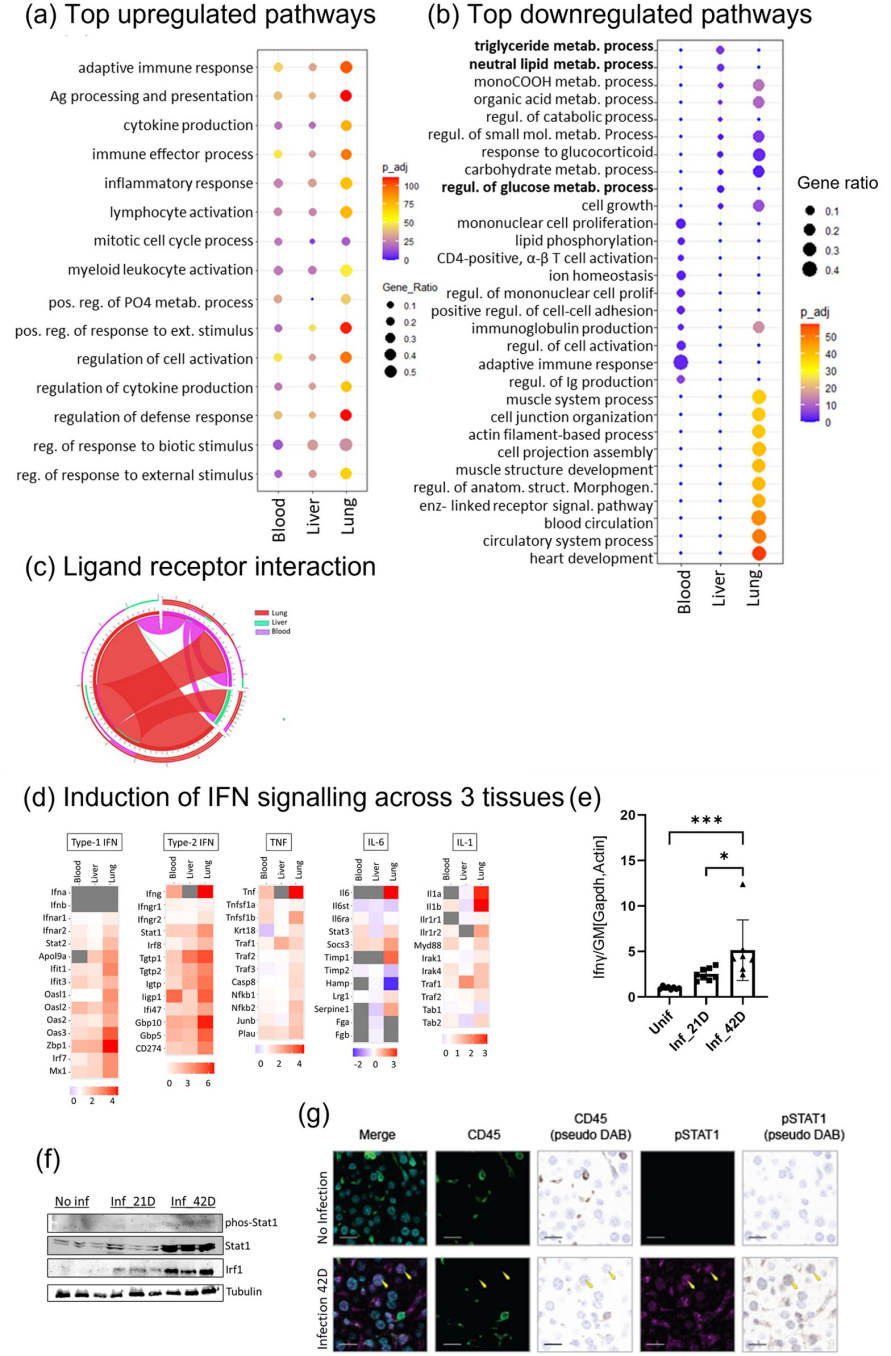
Aerosol infection of *Mycobacterium tuberculosis* shows tissue specific metabolism changes. (a) Livers (in house), lung (GSE137092) and blood (GSE137092) RNAseq data was compared between infected (42 day) and uninfected mice (n = 4–5) for non redundant pathways enriched by upregulated gene (FC 1.5, *padj <0*.*05*; upregulated genes in liver n = 520; lung n = 2862; blood n = 1207). (b) Non redundant pathways enriched by downregulated gene (FC 1.5, *padj<0*.*05*, downregulated genes in liver n = 163; lung n = 3450; blood n = 664). Pathways uniquely enriched in liver are highlighted as bold. (c) Ligand-receptor analysis shows interactions between lung, liver and blood of 42 day infected mice. Colour of the line represents each tissue and thickness represents number of genes. The outer arc covering each tissue represents number of overall interactions covering the specific tissue with others. The middle arch represents the number of the receptors in the covering tissue that have ligands in the same and other 2 tissues. The inner most arc represents number of ligands of the covering tissue that have receptors in same and other 2 tissues. (d) Inflammatory cytokine gene expression (fold change in colour-scale, gene not quantified due to below threshold in the transcriptomics data matrix i.e., <1 cpm are presented in grey). (e) Interferon gamma mRNA level quantified using RT-PCR analysis. (f) Western blot analysis of interferon signalling proteins in the bulk liver tissue of uninfected and infected mice. (g) Histochemical analysis of liver tissue of uninfected and 42 day infected mice of phospho-Stat-1 (magenta) in CD45 (green) positive and negative cells, nuclear stain (DAPI-blue) and pseudo-DAB in brown. Panels (f) and (g) are representative of 2 independent experiments (n = 5–6 per group per experiment).

The modest and contained infection in the liver, the observed infection-induced change in expression of genes and proteins (Fig 1) and the fact that immune signals influence metabolic function in a tissue and organ specific manner [25], suggests a systemic effect from the lung acting on the liver. To examine systemic transcriptomic changes in Mtb-infected mice, we compared the data for liver (in house), with data sets from lung and blood from the same mouse strains infected with the same bacterial strain via the same route and with similar bacterial burdens from the public database. We found that upregulation of immune-related pathways in response to Mtb infection occurred similarly in liver, lung, and blood (Fig 2a). In contrast, the downregulated pathways were different between tissues, with glucose metabolism, triglycerides, and natural lipid metabolism pathways being downregulated in the liver (Fig 2b). The downregulated pathways in the lung represent circulation, heart/muscle development and morphogenesis and those in the blood represent cellular proliferation, activation, and some immune-related pathways (Fig 2b). As the liver plays a pivotal role in glucose synthesis and in lipid synthesis and packaging, the unique reduction in the lipid and carbohydrate metabolism pathways in the liver (Fig 2b) will have significance to health. These data show that a predominantly pulmonary infection induces immune signalling across organs, while there is organ specific down regulation of metabolic pathways in the liver that may compromise health and require investigation.

To determine the potential for endocrine-like behaviour systemically, we carried out a ligand-receptor connectivity analysis [26] and found a well-connected ligand-receptor network is observed between lung, liver and blood with the liver expressing receptors for ligands differentially expressed in both lung and blood cells (Fig 2c), (Gene and interaction list in S4 Data). Among the three organs, the lung expresses the highest number of ligands that potentially bind to receptors within the lung, blood, and liver. Only one ligand is differentially expressed in the liver and has a receptor in the lung; this is C1qa, which is a known circulatory marker of human TB [27,28]. These data show that there is an inter organ network of gene expression which has the potential to mediate significant changes in organ specific behaviour in a systemic manner.

To determine which immune pathways may be mediating systemic effects, we evaluated the expression levels of genes involved in common immune/inflammatory pathway over all tissue gene sets. IL-6 is known to trigger acute phase proteins and complement factor release in the liver during inflammation [29], however, activation of IL-6 pathways is not observed in the livers of Mtb-infected mice at this relatively early time point (day 42) (Fig 2d). In contrast, target genes of both type I (alpha, beta) and type II (gamma) interferon (type I/II IFN) pathways are elevated in all three tissues examined (Fig 2d). Local production of interferons in the liver is negligible by transcriptomics analysis, although increased local production of mRNA for IFNγ can be seen (Fig 2e) and there is an increased frequency of activated CD4 and CD8 cells within the infected liver (S1B Fig) thus, activation of interferon signalling pathways within the liver could be both local and endocrine. To confirm that the observed transcriptional differences were represented at the protein level in the liver, we compared the level of IFN-induced signalling proteins and found both Stat-1 and Irf-1 were more highly expressed in the livers of mice infected for 21 and 42 days (Fig 2f). While relative increase in phosphorylation of Stat-1 is not observed, the translational increase of Stat-1 indicates IFN -induced signalling in the infected liver [30]. To determine whether IFN signalling was occurring in hepatocytes, we interrogated the level and location of phosphorylated Stat-1 in liver tissues by immunohistochemistry. We found that phosphorylated Stat-1 was colocalizing with nuclear staining in both CD45 positive and CD45 negative cells in infected but not uninfected liver tissue (Fig 2g). The nuclear morphology of the phosphorylated Stat-1 positive, CD45 negative cells is representative of hepatocytes. These data confirm the transcriptional evidence demonstrating that IFN-mediated signalling occurring in the liver, and specifically in hepatocytes, of Mtb infected mice.

### Mtb infection influences glycolysis and gluconeogenesis in the liver of infected animals

**Fig 3:**
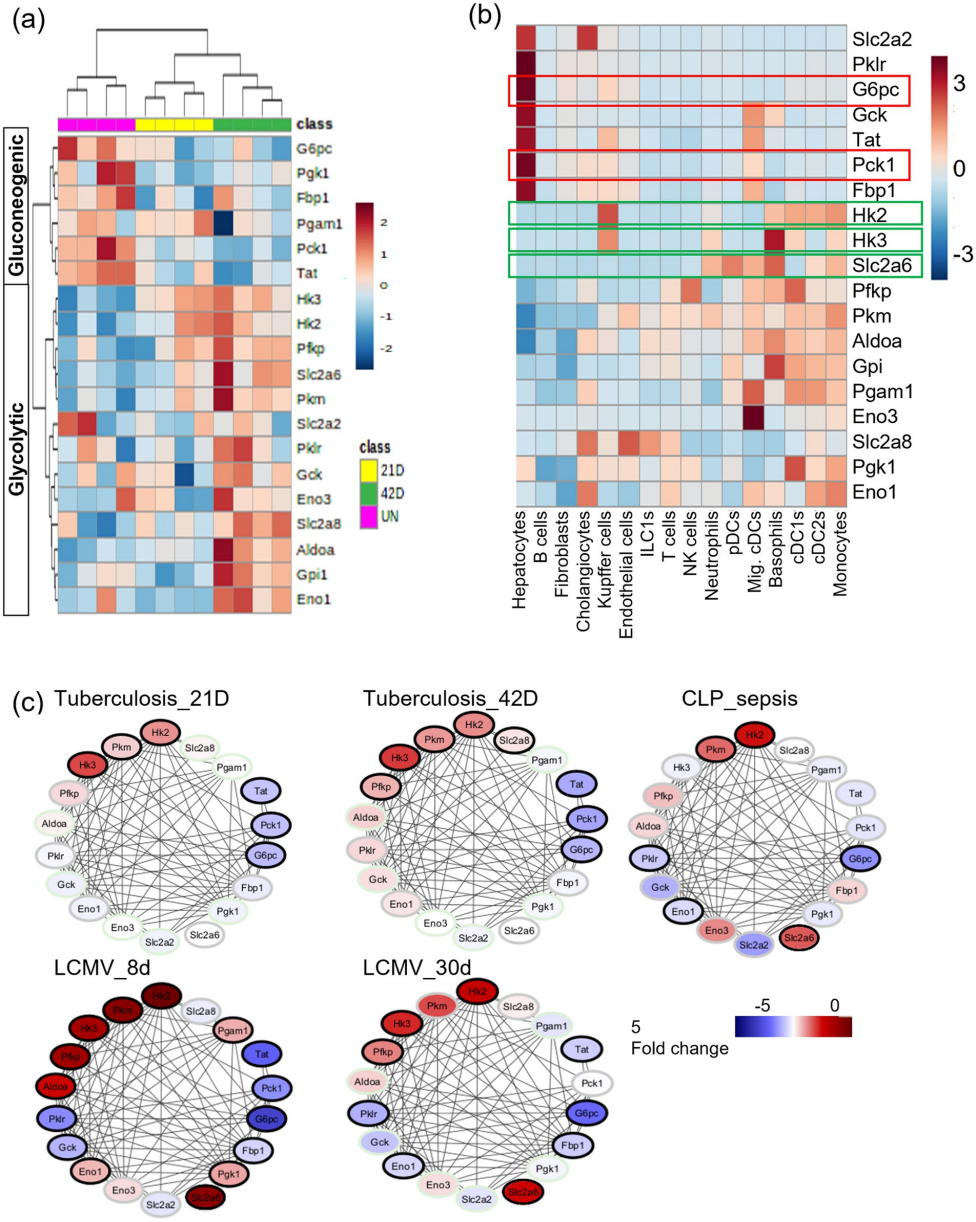
Pulmonary TB suppresses gluconeogenic gene expression in the liver. (a) Hierarchical clustering of genes involved in glycolytic and gluconeogenic gene expression. (b) Single cell RNAseq analysis of liver cells from GSE192742 to deconvolute the glycolytic and gluconeogenic genes from Fig 3a. (green boxes: upregulated, red boxes: downregulated). (c) Differential expression of gluconeogenic and glycolytic genes in the livers of aerosol Mtb infected mice (Tuberculosis_21D and Tuberculosis _42D) compared to the differential expression of the same genes in acute (day 8) and chronic (day 30) LCMV clone 13 infection (LCMV_day 8 and LCMV_day 30) and of these genes in an acute model of sepsis (CLP_sepsis). The colour of each node represents fold changes and the fold changes that are statistically significant p_adj_ <0.05 are represented with bold black margin.

Lipid and glucose metabolism pathways were both negatively impacted at gene and protein level in the livers of Mtb-infected mice reflecting the markers of liver dysregulation observed in patients with TB (Figs 1 and 2) (S2 Table shows carbohydrate pathways). To identify investigable pathways associated with Mtb infection, we undertook unsupervised hierarchical clustering with daughter gene sets from the lipid and glucose pathways identified by the cluster analysis of the liver transcriptome of uninfected and Mtb-infected mice. The lipid metabolism pathway daughter gene sets did not directly link to the experimental groups, likely reflecting the complex nature of lipid metabolism in the liver. In contrast, the liver genes best able to define infection-related clustering (uninfected, D21, and D42) are those for glycolysis and gluconeogenesis pathways including the genes for the rate limiting enzymes of gluconeogenesis, glucose 6 phosphatase (G6pc) and phosphoenolpyruvate carboxykinase 1 (Pck1). Importantly, genes comprising the glycolytic pathway were elevated in the livers of infected mice, while genes involved in gluconeogenesis were reduced (Fig 3a). Gluconeogenesis is considered to be the reverse of glycolysis [31] however, both processes could be happening independently within different cell types. To determine whether different cell types were contributing to the observed gene expression differences following Mtb infection, we used previously published single cell analysis of liver tissue to identify genes likely to be expressed only in hepatocytes. We found that the downregulated genes for Pck1 and G6pc (Fig 3a), which are rate limiting for gluconeogenesis, are specifically only expressed in hepatocytes in liver tissue (Fig 3b, red boxes). Tyrosine aminotransferase (Tat), which is a down regulated gluconeogenesis gene [32] (Fig 3a) is also expressed primarily in hepatocytes (Fig 3b). The upregulated glycolysis pathway genes such as solute carrier family 2 member 6—a glucose transporter (Slc2a6), hexokinases 2 and 3 (Hk2 and Hk3), and pyruvate kinase (Pkm) (Fig 3a) were found to be expressed primarily in immune cell types in liver tissue (Fig 3b green boxes). Importantly, glycolytic genes which are expressed in hepatocytes such as solute carrier family 2 member 2 (Slc2a2), glucokinase (GcK), pyruvate kinase (Pklr) did not vary during infection (Fig 3a and 3b). These data support the hypothesis that glycolysis is increased in the infiltrating immune cells whereas gluconeogenesis is significantly reduced in the hepatocytes.

To investigate whether the difference in glycolytic and gluconeogenic gene expression in Mtb infection is a universal response to stress or may be specific to TB, we compared the differential gene expression induced by Mtb in the liver with that of LCMV clone 13 induced gene expression changes in the liver over time (GSE118703) as well as with a more acute model of infection-induced stress, the caecal ligation and puncture induced sepsis (CLP-Sepsis) (GSE167127). By comparing the gene expression changes across these three models we observed a similar pattern of immune cell related glycolytic gene expression in all the three conditions. There was a variable pattern of reduced expression of hepatocyte specific glycolytic genes Pklr, Gck, Slc2a2 expression in both LCMV and in CLP-sepsis model although Pklr was reduced in all models (Fig 3c). Such differences may be due to higher hepatic pathogen burden and inflammation in both these models compared to Mtb infection. Suppression of gluconeogenic gene G6pc was consistent among all these infection cases, however, reduction of Pck1 was not observed in the LCMV resolving phase and in CLP-sepsis. These data support the hypothesis that Mtb drives a unique pattern of glucose dysregulation, while other infections stress the liver differently and exhibit distinct effects on glycolytic and gluconeogenic gene expression in the liver.

### Mtb infection compromises phosphorylation of CREB and type I and II IFN contribute to suppression of gluconeogenesis

**Fig 4:**
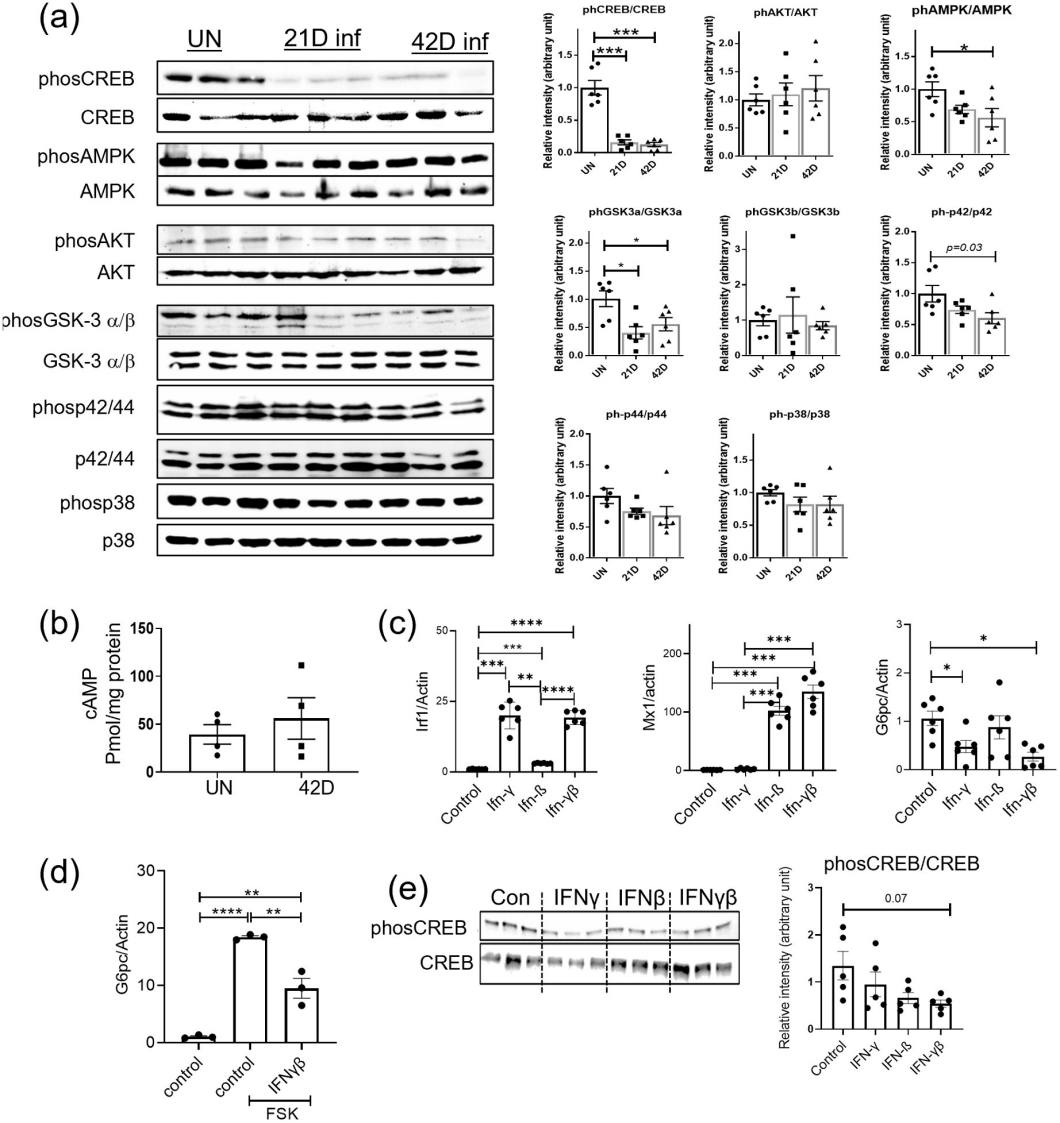
Mtb infection suppresses phosphorylation of CREB, a gluconeogenesis controlling transcription factor and interferon treatment compromises gluconeogenesis gene expression and protein phosphorylation in the hepatocyte. (a) Phosphorylation of proteins involved in glucose metabolism in the livers of uninfected (UN) and aerosol Mtb infected mice (21D inf, 42D inf) (blot shows n = 3, with combined intensity measurements (n = 6) shown in panels at right of blots). (b) cAMP level in livers of uninfected (UN) and Mtb aerosol infected (42D) mice. (c) Hepatocyte cell line HepG2, was cultured with IFN-γ (1200u/ml), IFN-β (1000U/ml) or a combination of both (IFN-γβ) for 16hrs and interferon signalling mRNA i.e., Irf1 and Mx1 and gluconeogenic mRNA i.e., G6pc quantified using qRT-PCR (n = 6). (d) Hepatocyte cell line, HepG2 was left untreated (control) or was treated with gluconeogenesis inducer forskolin (FSK) for 16 hrs in the absence (control) or presence of a combination of IFNγ (1200u/ml) and IFNβ (1000U/ml) (IFNγβ) and G6pc gene expression was quantified. (e) Hepatocyte cell line, HepG2 was incubated with IFN-γ (1200u/ml), IFN-β (1000U/ml) or a combination of both (IFN-γβ) for 16hrs and the level of phosphorylated CREB determined by Western bot (n = 3 in blot shown), intensity of phosphorylation band n = 6. For western blot and RT-PCR at least 2 independent experiments (n = 3 from each) were used for quantification. Anova and Student’s *t*-test were used for calculation of band intensity in western blot and mRNA relative level in RT-PCR. *<0.05, **<0.01, ***<0.001, ****<0.0001.

To investigate how gluconeogenesis is being regulated during aerosol delivered Mtb in the mouse model, we compared the signalling pathways active in regulating gluconeogenesis in the liver of uninfected and Mtb infected mice. Gluconeogenesis is activated by glucagon through CREB phosphorylation upregulating Pck1 and G6pc expression and is supressed by insulin through AKT phosphorylation [33–35]. Recently mice with TB have been shown to have high circulating insulin [36], and so we compared the level of endogenous insulin signalling between uninfected and Mtb-infected mice by measuring the phosphorylation state of AKT (AKT Serine/Threonine Kinase) in the livers. We found no difference in AKT phosphorylation during Mtb infection (Fig 4a). In contrast, we found that phosphorylation of CREB (cAMP response element-binding protein), the driver of gluconeogenesis, was substantially and significantly reduced (Fig 4a). Suppression of rate limiting gluconeogenic enzyme expression and CREB phosphorylation is independent of gender and age of the mice (S3A–S3C Fig). These data suggest that in Mtb infection, CREB phosphorylation is heavily compromised, which in turn may supress gluconeogenic gene expression.

CREB phosphorylation can be mediated by glycogen synthase kinase 3α/β (GSK3α/β) dephosphorylation and by stress-induced mitogen-activated protein kinases (MAPK) p42/44 (ERK1/2) and p38 [37,38]. To determine whether these pathways could be involved in compromised CREB phosphorylation, we compared them in liver samples from uninfected and infected mice. We found that GSK3α is dephosphorylated in infected mice and that among the MAPKs, p42 phosphorylation was supressed in the liver by day 42 of infection (Fig 4a). CREB dephosphorylation can be mediated by activation of protein phosphatase PP1 and PP2A [39], but we did not observe a difference in gene expression of these phosphatases in our transcriptomics data (Sheet A in S2 Data). Hepatic cyclic AMP (cAMP), which promotes CREB phosphorylation was also not different between uninfected and infected liver (Fig 4b). Another mechanism of gluconeogenic gene suppression in malaria and sepsis is suppression of G6pc by iron generated by heme oxygenation, which is compensated by iron-sequestering ferritin H chain (FTH) [40,41]. However, we did not see any difference in the gene or protein expression level of FTH chain (Sheet A in S2 Data and Sheet A S3 Data). Together our liver-specific signalling analysis suggests that the observed reduction in CREB phosphorylation is likely mediated downstream of cAMP in infected mice.

Inflammatory molecules or cytokines such as LPS [42], TNFα [40,43], IL1β and IL-6 [43] suppress gluconeogenic gene expression, particularly G6pc, in animal models, primary hepatocytes and hepatocyte cell lines independent of insulin and/or glucagon stimulation. We did not however see upregulation of the signalling pathways for TNFα, IL-1β and IL-6 in the livers of Mtb-infected mice whereas both type I and type II IFN interferon signalling pathways were upregulated (Fig 2d). We also knew that type II IFN can supress CREB phosphorylation in macrophages [44]. We therefore investigated whether IFNγ and IFNβ impact gluconeogenic gene expression in the HepG2 hepatocyte cell line. We chose the HepG2 cell line because it is a human hepatocyte cell line known to respond to cytokines and which has also been used in glucose metabolism studies [40, 41]. Addition of IFNγ activated the type II IFN signalling gene, Irf1 and addition of IFNβ activated the type I IFN signalling gene, Mx1 in HepG2 cells. Co-treatment with IFNγ and IFNβ induced expression of both Irf1 and Mx1 (Fig 4c). We found that IFNγ and IFNβ co-treatment significantly reduced G6pc expression (Fig 4c). Addition of forskolin, an activator of gluconeogenesis, increased G6pc expression in the HepG2 cell line, which was reduced by co-treatment with IFNγ and IFNβ (Fig 4d). Addition of IFNγ and IFNβ together also showed a trend towards compromised CREB phosphorylation (p = 0.07) (Fig 4e), suggesting that these interferons are candidates for CREB-mediated reduction of gluconeogenic gene expression in the liver during pulmonary TB.

### Mtb infection compromises insulin responsiveness in mice and insulin resistance correlates to TB disease resolution and progression in humans

**Fig 5:**
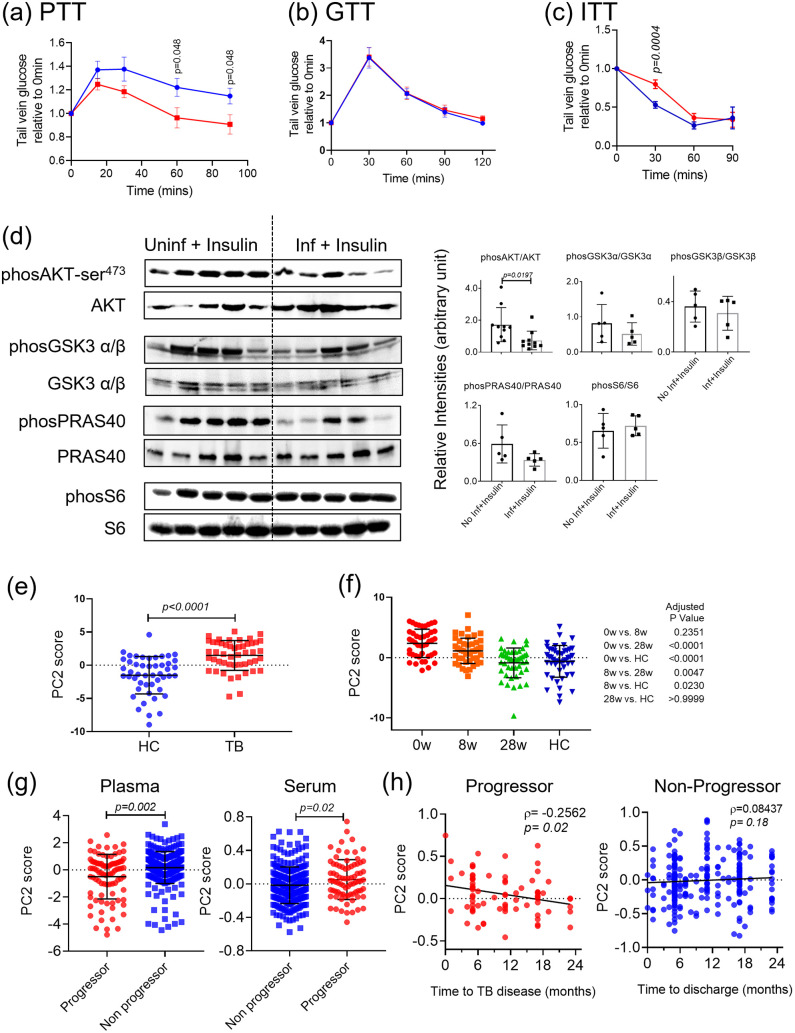
Mtb infection and TB progression are associated with insulin resistance. (a, b and c) Mice were left uninfected (control, blue symbols) or aerosol infected with Mtb (42d Inf, red symbols), fasted and then treated with either (a) pyruvate, (b) glucose or (c) insulin and the level of glucose in the blood determined over time (n = 6–8 from 2 independent experiment, age and gender matched mice) (a-c, multiple *t*-test with comparison between uninfected and infected for each time point from 2 independent experiments combined). (d) Western blot and densitometry quantification of phosphorylation of proteins in the livers of uninfected (No Inf) or aerosol Mtb infected (Inf) insulin injected mice (n = 5–10, from 2 independent experiment, age and gender matched mice, Student’s *t*-test was used for densitometric analysis). (e, f) Reanalysis of metabolomics data (Veiling et. al. 2019), comparing the BCAA related metabolite pattern (PCA, PC2 score) in plasma between healthy controls (HC) and untreated TB patients (TB) (e) and over time from baseline (0w) to 28 weeks of treatment (28w) and healthy controls (HC) (f). (g, h) Reanalysis of metabolomics data (GC6-74), from exposed individuals who either did (progressor, red symbols) or did not (non-progressor blue symbols) progress to TB over 24 months. (g) Direct comparison of BCAA related metabolite pattern (PCA, PC2 score) for both plasma and serum between progressor and non-progressor. (h) Correlation analysis between the time to progression to TB disease (left, progressor) or to discharge (right, non-progressor) and changes over time of BCAA-related metabolic pattern (PCA, PC2 score) in serum samples. (e-h, comparisons between groups- Mann Whitney (e, g) or Kruskal Wallis test (f) and Spearman correlation (ρ).

Because we observed a drop in gluconeogenic gene expression in the livers of Mtb infected mice, we wanted to determine if this had any phenotypic outcome. To test the ability of the liver to utilize new substrate for gluconeogenesis, we undertook a pyruvate tolerance test in overnight-fasted mice and found that infected mice were less able to maintain glucose production in response to exogenous substrate than were uninfected mice (Fig 5a) (AUC 15–90 mins infected vs uninfected is significantly different, *p* = 0.05, Mann Whitney). We also compared fasting glucose levels between non-infected and infected (day 42) mice and found no difference (S3D Fig), in agreement with previous reports [45, 46]. We then carried out a glucose tolerance test in overnight fasted mice and found no difference in the tail vein glucose level at any time point post glucose injection (Fig 5b). Comparing insulin sensitivity between infected and uninfected mice showed that exogenous insulin was slower to reduce blood glucose in the infected mice (Fig 5c) (AUC infected vs uninfected is significantly different, *p* = 0.0115, Mann Whitney). In confirmation of reduced systemic response to exogenous insulin, we found that infected mice also exhibited reduced hepatic AKT phosphorylation in response to exogenous insulin compared to uninfected mice (Fig 5d). There was also a trend toward reduced phosphorylation of GSK3b and PRAS40 (Fig 5d), which are downstream of AKT. Together these data suggest that aerosol infection with Mtb reduces gluconeogenesis in the liver and the ability of the host to respond to insulin both at the systemic level and at the level of the hepatocyte intracellular signalling pathways.

This observed impact of lung Mtb infection on the liver suggests that liver metabolism can be impacted when liver infection and inflammation is modest, relative to the lung. We wanted to investigate whether insulin resistance is also associated with TB disease and/or infection in humans and if TB treatment has any impact on the signs of insulin resistance. To do this, we have reanalysed metabolomics data sets from people with active TB who are undergoing treatment [47] and from people exposed to TB who have been monitored for progression after exposure [48]. We have analysed the metabolomics data using the branch chain amino acid (BCAA) related metabolic pattern defined by Newgard et al. [49]. Newgard et al. have shown that disrupted branched chain amino acid metabolism, as measured by a metabolite signature of altered circulating levels of branched-chain and aromatic amino acids and glycine, correlates well with HOMA-IR, a measure of insulin resistance [49,50]. We used a similar PCA based approach for dimension reduction of the amino acid metabolic markers in the published data sets and found in the TB treatment cohort that the BCAA signature can discriminate TB patients from healthy control (Fig 5e) and that the BCAA signature resolves towards that seen in healthy controls as treatment progresses (Fig 5f). Metabolomics analysis of a cohort of people exposed to TB also showed statistically different BCAA-related metabolite based PCA scores between those progressing to active TB (progressor) and those who remained disease free (non-progressor) (Fig 5g). Interestingly, changes in the BCAA-related PCA score from the serum samples showed a statistically significant temporal correlation with the time to TB diagnosis. Such correlation was not observed in serum samples of those who did not develop TB (Fig 5h). These data support the hypothesis that insulin resistance correlates with TB disease activity in both mice and humans.

## Discussion

The body is a complex system and responds to environmental cues with coordination between cells and organs and this coordination is essential in maintaining homeostasis. In pulmonary TB, the homeostasis between cells and organs is perturbed and can contribute to the observed systemic symptoms. Our data show that pulmonary TB can impact liver function in humans. We have also shown that while there is a relatively modest and contained bacterial burden and limited inflammation in the liver, we see significant immunological and metabolic transcriptomic and proteomic changes. Among the key changes we observed in the liver were upregulation of IFN signalling pathways and suppression of gluconeogenic gene expression and CREB phosphorylation (see model S4 Fig). These functional changes in the liver were accompanied by decreased capacity for gluconeogenesis and systemic and hepatic insulin sensitivity. Our subsequent metabolomics analysis of two TB cohorts—a treatment and a TB exposed cohort—demonstrated that metabolic markers of insulin resistance are also associated with active TB in humans. Together our data support the hypothesis that pulmonary Mtb infection results in dysregulated liver metabolic function and systemic and hepatic insulin resistance.

While our data are surprising due to the relatively modest infection level in the liver and the relatively early onset of metabolic effects in both mice and humans, infection is known to influence local and systemic homeostasis. Indeed, the liver plays a role key in maintaining health during systemic and hepatic infection with immunological signals influencing the ability of the liver to maintain homeostasis [51–53]. However, these previous studies were carried out with pathogens which are hepatotropic or systemic and so an impact on the liver was not surprising. In pulmonary TB and in the Mtb infection model we have used, the infection is neither hepatotropic nor systemic, with relatively modest inflammation in the liver and with most of the tissue being composed of hepatocytes. Hepatocytes are not classical immune cells; however, they express receptors for and respond to many cytokines with clear cytokine target genes being activated [54,55]. Such immune activation mechanisms in the hepatocyte, alongside activation of resident and infiltrating immune cells is likely to influence metabolic programming of the hepatocyte. Our data show for the first time that a localised and contained pulmonary Mtb infection has a significant impact on hepatic metabolic function. Our observation is supported by a recent study showing little to no bacterial burden in the adipose tissue of pulmonary Mtb infected mice despite clear inflammation-induced disruption of lipid metabolism and hypertrophy in adipocytes [46].

Our transcriptomics and proteomics analyses highlight the potential for the pulmonary immune response to influence the liver through the blood via a range of potential mediators. The clear induction of type I and type II IFN signalling pathways in the liver support the hypothesis that interferon related pathways are impacting liver function. While both lipid and glucose metabolic pathways were seen to be down regulated, we found that the key glucose metabolic genes were clearly aligned with infection, making these pathways directly investigable. While we focus here on glucose metabolism, we are also pursuing the impact on lipid metabolism, which requires more complex tools and interventions for definitive outcomes. The ‘Omics data highlighted changes to overall glucose metabolism while our focused analysis highlighted suppression of gluconeogenic gene expression in liver cells. This observation contrasts with the largely accepted working model that infection and inflammation promote hepatic gluconeogenesis to provide glucose to support immune function [56,57]. In fact infection-induced acute hyperglycaemia is seen mostly in extreme cases such as sepsis [51] and recent publications in both human and animal models show that hypoglycaemia can be an outcome of infection with concomitant suppression of hepatic gluconeogenic gene expression [40,41]. Indeed, a recent study has reported that both hypo- and hyper-glycaemia are seen in hospitalized Covid-19 patients, and both can be associated with increased risk of poor renal and cardiovascular outcomes [58].

Regardless of the impact of infection on glucose metabolism, the mechanisms whereby infection disrupts metabolism are ill defined. Our observation of a dramatic reduction of phosphorylation of CREB, a key transcription factor regulating gluconeogenesis is novel and prompts the need for in depth analysis of the pathways inducing this effect. Our investigations begin this analysis by demonstrating a trend towards CREB dephosphorylation being induced by type I and II IFN in hepatocytes. Both inflammatory and immune-related signalling molecules like LPS and TNF, can directly modulate hepatic gluconeogenic gene expression independently of classical metabolic mediators such as insulin, glucagon, catecholamine, and glucocorticoids [40,41,43]. By showing the ability of IFN to drive signalling in hepatocytes in vivo and a direct inhibitory effect of type I and II IFNs on expression of a key gluconeogenesis gene in hepatocytes, possibly via CREB, we add to the understanding of how immune mediators can reduce liver gluconeogenesis. As type II IFN is key to survival following Mtb infection and type I IFN is associated with poor outcomes, studies with hepatocytes unable to respond to either type I or II IFNs will be required to define their relative role in reduced gluconeogenesis in the hepatic tissue of pulmonary Mtb infected mice.

Regardless of the pathway by which gluconeogenesis is reduced, the observed reduction in gluconeogenesis must be balanced in the host, to ensure maintenance of peripheral glucose level. In this regard we found that Mtb infection results in reduced ability to convert pyruvate to glucose and reduced systemic and liver insulin sensitivity. This balance between glucose production and utilization may explain why the mice are able to maintain peripheral glucose levels at this level and time of infection. There are other reports of increased insulin resistance in Mtb infected mice [36] and of glucose intolerance early in infection in Mtb-infected guinea pigs [59]. Insulin resistance is also reported in mice infected with viral pathogens such as LCMV, MCMV and in mice treated with LPS [51,60]. The required balance between gluconeogenesis and insulin resistance may be mediated by immune signals as hepatocytes treated with TNF, LPS or type I/II IFN show attenuated insulin signalling [60–62]. These observations suggest that immune regulation of gluconeogenesis and insulin signalling occurs during infection, and it will be important to determine how this impacts long-term glucose homeostasis when pathogens, such as Mtb, are not cleared by the immune response.

TB diagnosis has been recently redefined by a consensus study that re-recognizes the important range of sub-clinical TB occurring worldwide [4] and our data is critical to the appreciation of the health impact this large proportion of individuals coping with TB. TB in humans often highlights altered glucose homeostasis with hyperglycaemia and insulin resistance being detected at the time of diagnosis. An important question raised by this observation is whether insulin resistance and/or the dysglycaemia seen in TB, is a normal result associated with infection or is a herald event indicating susceptibility to metabolic disease, such as diabetes [63]. Importantly, longitudinal analyses of hyperglycaemia and glycated haemoglobin show resolution during and after TB treatment in a section of patients, suggesting association between dysglycaemia and TB infection [64,65]. Our data showing reduced gluconeogenesis and insulin resistance as the immune response and inflammation begin to be expressed in infection suggests that glucose dysregulation is a product of infection and that if infection is not resolved then it could contribute to development of dysglycaemia. This is an important issue as the extent of undiagnosed subclinical TB is high [4] and this unappreciated immune pressure could be driving susceptible individuals towards diabetes and thereby contributing to the increase in diabetes in TB endemic countries [66]. Our reanalysis of metabolomics data from people developing TB and people being treated for TB, demonstrates that active infection is associated with an insulin resistance signature involving BCAA metabolism. BCAA metabolism is associated with insulin resistance and diabetes and catabolism of BCAA is a therapeutic target for diabetes [49,67–69]. BCAA also activates mTORc a key regulator of glucose metabolism and is a target of the key diabetic drug metformin [69,70]. A potential role of mTORc2 in mediating systemic inflammation during Mtb infection has been shown in mTORc2 deficient mice aerosol infected with Mtb, who fail to develop inflammation in their adipose tissue [46]. These data may serve to explain the observations that people who are latently infected with Mtb have higher average insulin resistance compared to non-infected controls despite equivalent glycosylated haemoglobin and body mass index [71]. Similarly, latent Mtb infection is associated with increased risk of diabetes in a retrospective cohort study [72]. These observations, in combination with our data, support the relevance of screening for insulin resistance in both latent and active TB as well as in TB exposed cohorts. This screening will allow better definition of the role of TB in driving the trajectory towards diabetes in these cohorts. In addition, it might be helpful to include tests for TB exposure in those undergoing diagnostic assessment for diabetes.

In summary we have shown that despite the liver not being a primary site of infection, both lipid and glucose metabolic gene and protein expression are perturbed during TB. We propose that this perturbation is initiated by infection-induced type I and type II IFN signalling, which drives dephosphorylation of CREB and down regulation of gluconeogenic gene expression. These changes are then reflected in compromised local and systemic insulin responsiveness which we think is required to balance gluconeogenesis suppression and insulin mediated peripheral glucose maintenance during early stage of infection. It is therefore critical that we understand the subtle and not so subtle impact of this infection thereby allowing precision care for those exposed to TB worldwide. We propose, at a minimum, that metabolic screening for insulin resistance and prediabetes should be a critical part of any TB screening programme.

## Materials and method

### Ethics statement

All procedures involving live animals were performed under UK Home Office license to AMC (P6DCE1A76) under an establishment license to University of Leicester (UoL) (X1798C4D2), the UoL Animal Welfare and Ethical Review Board approved the project license prior to submission to the Home Office.

### Secondary analysis of biochemical parameters from clinical studies

Liver function and blood lipid profiles from case-control studies involving drug-naïve adult TB patients were retrieved from PubMed. It was not a systemic review however PubMed was searched with the keywords ‘tuberculosis’, ‘biochemical parameter’, ‘cholesterol’, ‘liver function’, ‘albumin’, ‘AST/ALT’, ‘SGOT/SGPT’. Unit values of each parameter (e.g., mg/dL to mM) were uniformly curated. Mean values were used for calculation of percentage difference between healthy control and TB. When published data was presented as median with range, median was converted to mean using the parameter of median, range and sample size when available according to Hozo et al [73].

### Animal

C57BL/6J were originally obtained from The Jackson Laboratory (Bar Harbor, Maine, US) through Charles River and bred in house at the University of Leicester under license.

### Study design

Experimental design was based on a two-group (uninfected and infected) study cohort in which the experimental unit was defined as a single mouse identified by ear punch, with the two groups housed together. Inclusion and exclusion criteria: any animal showing signs of unexpected illness during procedure was excluded; all animals not specifically excluded were included in experimental design. Individual mice were randomly assigned to either of the groups and remained identifiable by ear punch throughout. Staff were blinded to mouse group during interventions. Bias was avoided by obscuring of groups and randomization. Groups included male and female mice between 6–8 weeks of age at the start of the experiment. Experimental intervention was infection, fasting, delivery of pyruvate, glucose or insulin and sequential tail bleeding. Interventions were undertaken at the same time of day for both groups of mice.

### Isolation of hepatic non-parenchymal cells and flow cytometry

Flow cytometry analysis was carried out to characterise the immune cells from the liver of uninfected and infected cell. Non parenchymal cells were isolated from the liver tissue using ex-vivo enzyme digestion method using a liver dissociation kit (Miltenyi Biotech, Germany) as per manufacturer’s protocol. Briefly, whole liver was dissected out from the animal and the gall bladder was removed aseptically. Liver was digested in the digestion mixture and homogenised in the GentleMACS C-tubes and non-parenchymal cells were purified following centrifugation. Cells were treated with Aqua live Dead stain prior to staining with fluorochrome conjugated antibodies. Stained cells were fixed in buffered formalin overnight and analysed in BD FACSCelesta instrument (BD Bioscience, USA). Raw data were analysed using FlowJo (BD Bioscience, USA). Antibodies used for the analysis are CD8 (BioLegend #100743), CD19 (Invitrogen #11-0193-82), CD69 (Invitrogen #12-0691-82), TCRβ (Invitrogen #45-5961-82), CD4, (BD #557956), CD44 (eBioscience 47-00441-82), CD11b (BD #563015), Ly6G (BD #740554), CD11c (BD #563735), Siglec-F (BD # 562757) and CD45 (BD #559864).

### Cell lines

HepG2 cells were kindly gifted by Prof Karl Herbert, University of Leicester. Cells were maintained in low glucose DMEM with 10% FCS. For cytokine treatment, serum starved cells were treated with IFNγ (1200u/ml, PeproTech) and/or IFNβ (1200u/ml, PeproTech) in DMEM with 1% FCS for 16 hours.

### Infection

Aerosol infection was carried out as previously described [16]. Briefly, the animals were infected with the H37Rv strain of Mtb through the aerosol route. A self-contained bespoke aerosol chamber (Walker Safety Cabinets Ltd., Glossop, UK) based on the ‘jet in air’ venturi nebulizer was used to deliver approximately 100 colony forming units (CFU) into each mouse lung. Infected mice were killed by anaesthetic overdose and organs were aseptically excised and individually homogenized in saline in M tubes using the Miltenyi Biotec gentleMACS Dissociator (Miltenyi Biotec, Bisley, UK). Homogenates were serially diluted in sterile saline and plated on nutrient 7H11 agar (Sigma-Aldrich). CFU were counted after 3 weeks of incubation at 37 °C [74].

### Organ harvesting and sample processing

For biochemical and molecular analysis, organs were directly harvested and snap frozen in dry ice and then stored at -80°C. For protein extraction ~100 mg tissue blocks were homogenised in ice cold RIPA buffer with protease inhibitor (cOmplete EDTA-free tablet, Merck) and phosphatase inhibitor (PhosSTOP, Merck) using the GentleMACS homogeniser. Protein lysate was centrifuged at 16,000g for 15 mins at 4°C and passed through 0.2μm PES syringe filter for sterilization. Protein quantification was carried out using the Bicinchoninic acid (BCA) assay (Pierce, Thermo Fisher, UK). For RNA extraction ~50 mg tissue samples were homogenised in 1 ml Trizol using GentleMACS homogenizer immediately post-harvest and then stored at -80°C. RNA was purified using RNeasy mini kit (Qaigen). Isolated RNAs from each sample were treated with DNAse (TURBO DNase (2 U/μL), Thermo Fisher Scientific) to remove DNA contamination and DNAse treated samples were further cleaned-up with RNeasy mini kit. RNAs were quantified in a NanoDrop and RNA integrity was assessed in Agilent Bioanalyzer 2100 system.

### RNAseq

Purified and DNAse treated RNA samples were assessed for purity, degradation/ contamination and integrity were checked using NanoDrop, agarose gel electrophoresis and Agilent 2100 respectively. Library concentration was first quantified using a Qubit 2.0 fluorometer (Life Technologies). Insert size was checked on an Agilent 2100 and quantified using quantitative PCR (Q-PCR). Sequencing libraries were generated using NEBNext UltraTM RNA Library Prep Kit for Illumina (NEB, USA) following manufacturer’s recommendations and index codes were added to attribute sequences to each sample. Briefly, mRNA was purified from total RNA using poly-T oligo-attached magnetic beads. Fragmentation was carried out using divalent cations under elevated temperature in NEB Next First Strand Synthesis Reaction Buffer (5X). First strand cDNA was synthesized using random hexamer primer and M-MuLV Reverse Transcriptase (RNase H-). Second strand cDNA synthesis was subsequently performed using DNA Polymerase I and RNase H. Remaining overhangs were converted into blunt ends via exonuclease/polymerase activities. After adenylation of 3’ ends of DNA fragments, NEBNext Adaptor with hairpin loop structure were ligated to prepare for hybridization. To select cDNA fragments of preferentially 150~200 bp in length, the library fragments were purified with AMPure XP system (Beckman Coulter, Beverly, USA). Then 3 μl USER Enzyme (NEB, USA) was used with size-selected, adaptor ligated cDNA at 37 °C for 15 min followed by 5 min at 95 °C before PCR. Then PCR was performed with Phusion High-Fidelity DNA polymerase, Universal PCR primers and Index (X) Primer. PCR products were purified (AMPure XP system) and library quality was assessed on the Agilent 2100.

### RNAseq data analysis

Raw data were analysed using Galaxy platform [75].The raw data were uploaded and pre-processed using Trimmomatic tool. The adapters were removed and raw reads below the 25 bases long and Phred score of below 30 were removed. For alignment, HISAT2 v2.0.4 with default settings and mouse reference genome mm10/GRCm38 was used. Following mapping and alignment, the reads obtained were quantified using HTSeq-count to obtain the gene-level counts. For HTseq-count parameters, for strandedness, the non-stranded option was selected. For the rest of the parameters default settings were used. For differential gene expression analysis, iDep.94 was used [76]. Briefly, HTseq-count data were loaded and pre-processed to keep genes of more than 1cpm in at least 3 libraries. Pre-processed count data were transformed using EdgeR. To identify Differential Expressed Genes (DEGs), DEseq2 method was used. For analysis of dataset from public repository (Gene Expression Omnibus, NCBI), SRA files were directly uploaded in the Galaxy platform and reads were extracted using FastQ format. The data were analysed the same way as described for primary liver RNAseq data. Dataset GSE137092 (TB-blood), GSE137093 (TB-lung) were selected as the mouse strain, pathogen strain, route of infection, CFU dose, and time of organ harvest post infection were either identical (mouse and pathogen strain, route, harvest) or very similar (dose 100CFU versus 100-450CFU) to the in-house experimental protocols that generated the livers for analysis. For LCMV-clone13 and caecal ligation and puncture-sepsis analysis dataset GSE118703 and GSE167127 respectively were extracted from the Gene Expression Omnibus, NCBI as raw files and analysed as described above.

### Ligand receptor analysis using RNAseq data

Ligand receptor analysis was done as described in Kadoki et. al., [26]. Briefly, mouse ligand-receptor pairs were retrieved for the CellTalkDB database [77]. For selection of ligand to be included in the study, the parameters were upregulated gene (*p*_*adj*_ <0.05) with minimum 50 cpm and their corresponding receptors having at least 50 cpm in expression value in any of the tissues.

### Proteomics

Precipitated proteins (approximately 500 μg) were solubilized by adding 100 mM TEAB and sonicating for 30 minutes. Tandem Mass Tag (TMT) labelling was performed according to the manufacturer’s protocol (https://www.thermofisher.com/order/catalog/product/90110). For the total proteome each digested protein sample was labelled with 9 of the TMT tags from a 10-plex kit (126, 127N, 127C, 128N, 128C, 129N, 130N and 130C). Post-labelling, samples were combined and cleaned on Sep-Pak C18 cartridge, dried and dissolved in 20mM ammonium formate (pH 10). The solution was then pipetted into a sample vial and placed into the autosampler of a Waters Acquity UPLC pump (Waters Corporation, Milford MA) for high pH reverse-phase fractionation.

The following LC conditions were used for the fractionation of the TMT total proteome samples: desalted peptides were resuspended in 0.1 mL 20mM ammonium formate (pH 10.0) + 4% (v/v) acetonitrile. Peptides were loaded onto an Acquity bridged ethyl hybrid C18 UPLC column (Waters; 2.1 mm i.d. x 150 mm, 1.7 μm particle size), and profiled with a linear gradient of 5–60% acetonitrile + 20 mM ammonium formate (pH10.0) over 60 minutes, at a flow-rate of 0.25 mL/min. Chromatographic performance was monitored by sampling eluate with a diode array detector (Acquity UPLC, Waters) scanning between wavelengths of 200 and 400 nm. Samples were collected in 1-minute increments and reduced to dryness by vacuum centrifugation.

Dried fractions from the total proteome of the high pH reverse-phase separations were re-suspended in 30 mL of 0.1% formic acid and placed into a glass vial. 1 mL of each fraction was injected by the HPLC autosampler and separated by the LC method detailed below. A total of 24 fractions were analysed by LC-MS/MS for the total proteome.

LC-MS/MS experiments were performed using a Dionex Ultimate 3000 RSLC nanoUPLC (Thermo Fisher Scientific Inc, Waltham, MA, USA) system and a Fusion Lumos Orbitrap mass spectrometer (Thermo Fisher Scientific Inc, Waltham, MA, USA). Peptides were loaded onto a pre-column (Thermo Scientific PepMap 100 C18, 5mm particle size, 100A pore size, 300 μm i.d. x 5mm length) from the Ultimate 3000 auto-sampler with 0.1% formic acid for 3 minutes at a flow rate of 10 mL/min. After this period, the column valve was switched to allow elution of peptides from the pre-column onto the analytical column. Separation of peptides was performed by C18 reverse-phase chromatography at a flow rate of 300 nL/min using a Thermo Scientific reverse-phase nano Easy-spray column (Thermo Scientific PepMap C18, 2μm particle size, 100A pore size, 75 μm internal diameter x 50cm length). Solvent A was water with 0.1% formic acid and solvent B was 80% acetonitrile, 20% water with 0.1% formic acid. The linear gradient employed was 3–40% B in 93 minutes. (Total LC run time was 120 mins including a high organic wash step and column re-equilibration).

The eluted peptides from the C18 column LC eluant were sprayed into the mass spectrometer by means of an Easy-Spray source (Thermo Fisher Scientific Inc.). All m/z values of eluting peptide ions were measured in an Orbitrap mass analyser, set at a resolution of 120,000 and were scanned between m/z 380–1500 Da. Data dependent MS/MS scans (Top Speed) were employed to automatically isolate and fragment precursor ions by collision-induced dissociation (CID, Normalised Collision Energy (NCE): 35%) which were analysed in the linear ion trap. Singly charged ions and ions with unassigned charge states were excluded from being selected for MS/MS and a dynamic exclusion window of 70 seconds was employed. The top 10 most abundant fragment ions from each MS/MS event were then selected for a further stage of fragmentation by Synchronous Precursor Selection (SPS) MS3 (1) in the HCD high energy collision cell using HCD (High energy Collisional Dissociation, (NCE: 65%). The m/z values and relative abundances of each reporter ion and all fragments (mass range from 100–500 Da) in each MS3 step were measured in the Orbitrap analyser, which was set at a resolution of 60,000. This was performed in cycles of 10 MS3 events before the Lumos instrument reverted to scanning the m/z ratios of the intact peptide ions and the cycle continued.

MaxQuant software (ver. 1.6.16.0) integrated Andromeda search engine was used for pre-processing of the raw LC-MS/MS file [78]. Default settings for the first and the main search tolerance were used i.e., 20ppm and 4.5 ppm respectively. The TMT data was searched based on 0.5 Da of a product mass tolerance with a maximum of two missed tryptic digestions. For fixed modification carbamidomethylation of cysteine residues was specified and for variable modification acetylation of lysine residues and oxidation of methionine residues were selected. The false discovery rate (FDR), set at 1% for peptide identifications, was determined based on a reverse nonsense version of the original database. The MaxQuant output were further processed for statistical analysis using Perseus (ver.1.6.1.4) [79] to identify the statistically significant protein.

### Pathway enrichment

Pathway enrichment analyses of both transcriptomics and proteomics were performed using g:Profiler (https://biit.cs.ut.ee/gprofiler) [80]. Transcriptomics and proteomics data were subjected to cluster analysis and gene/proteins representing each cluster were used as input list and hypergeometric distribution tests was conducted to estimate the *p*-value. Multiple testing correction was carried out for *p*-value adjustment by an inbuilt algorithm i.e. g:SCS (set counts and sizes). Redundant pathway terms were removed by ReVIGO (21789182). For loss and gain of hepatic gene identity, genes and proteins are ranked as per the fold change and significant differences [Rank = (-log10p-value) x fold change] and assessed using Gene Set Enrichment Analysis (GSEA). For hepatocyte specific gene set was derived for the PanglaoDB [81] and run with a permutation of 1000.

### Single Cell RNAseq data analysis

Single cell RNAseq data from healthy mouse liver were downloaded from the publicly available dataset GSE192742 for deconvolution of cellular gene expression of the glycolytic/gluconeogenic genes. Annotation files for the same study were downloaded from the https://www.livercellatlas.org/ (accessed on 02/08/2023).

### PAGE and Western blot

Equal amounts of protein lysate were treated with Laemmli buffer and proteins were resolved in 10 or 12% Polyacrylamide gel. Resolved protein gels were transferred onto methanol activated PVDF membranes for 90 mins using wet transfer method or semi dry turbo transfer method. Membranes were blocked in 3% BSA in TBST (20mM Tris, 150mM NaCl, 0.05% Tween 20). Blocked membranes were treated with primary antibody overnight followed by TBST wash. Washed membranes were treated with horseradish peroxidase (HRP) linked secondary antibody for 1.5 hours (CST) and visualised with ECL solution (BioRad) in Chemidoc system (BioRad). Densitometry analysis was carried out using ImageLab software (BioRad). Antibody used were phospho-CREB (CST9198), CREB (CST9197), phospho-AMPK (CST2535), AMPK (CST5832), phospho-Akt (CST4060), Akt (CST4691), phospho-GSK-3 (CST9331), GSK-3 (CST5676), Phospho-ERK1/2 (p42/44) (CST4695), ERK1/2 (CST4370), phospho-p38 (CST4511), p38 (CST8690), phosphoPRAS40 (CST5936), PRAS40 (CST2610), PhosphoS6 ribosomal protein (CST2215), S6-ribosomal protein (CST2217), anti-rabbit HRP (CST7074).

### qRT-PCR

cDNA was synthesised from equal amounts of RNA using RevertAid First strand cDNA synthesis kit using manufacturer’s protocol (Thermo Scientific, UK). A volume of 1 μl of cDNA sample was used for qRT-PCR using FastSybr Green Master Mix (Thermo Fisher, UK) amplified in Fast7500 (Applied Biosystem) or LightCycler96 (Roche) system. Primers used for the assays are mouse-G6pc_Forward: ACACCGACTACTACAGCAACA G, mouse-G6pc_Reverse: CCTCGAAAGATAGCAAGAGTAG, mouse-Pck1_Forward: CATATGCTGATCCTGGGCATAAC, mouse_Gapdh_Forward: TGACCACAGTCCATGCCATC, mouse_Gapdh_Reverse: GAC GGA CAC ATT GGG GGT AG, mouse_Actin_Forward: CTG GCT CCT AGC ACC ATG AAG AT, mouse_Actin_Reverse: GGT GGA CAG TGA GGC CAG GAT, human_G6pc_Forward: ACT GTG CAT ACA TGT TCA TC, human_G6pc_Reverse: TGA ATG TTT TGA CCT AGT GC, human_Actin_Forward: ACC AAC TGG GAC GAC ATG GAG AAA, human_Actin_Reverse: ATA GCA CAG CCT GGA TAG CAA CG.

### Immunohistochemistry and Multiplex immunofluorescent (mIF) staining

Paraffin-embedded liver tissue sections on slides were stained as previously described using the Novolink Polymer Detection System (Leica BioSystems). Briefly, they were heated to 65°C, dewaxed and rehydrated in graded IMS and rinsed with tap water. Slides then underwent antigen-retrieval in citrate buffer (pH 6.0), were washed and incubated with protein blocking agent, probed with anti-CD45 antibody overnight (CST70257), washed, and incubated with Novolink Polymer Detection system. Slides were washed in PBS incubated with diaminobenzidine, counter stained with haematoxylin, dehydrated in graded IMS, cleared in xylene, and mounted in DPX.

Multiplex IF (mIF) was performed as previously described in detail [82] and antibodies were paired with Opal fluorophores (Akoya Biosciences, UK) as follows: CD45 (CST#70257, 1:200; Opal520), pStat-1 (CST#9167, 1:500; Opal690). Whole slides were scanned using a PhenoImagerHT (Akoya Biosciences, UK). Phenochart was used to visualise whole slide scans and to generate pseudo-diaminobenzidine (DAB) images for each antibody.

### cAMP level

The cAMP level from frozen liver tissue was measured using cAMP Elisa kit (Enzo, Life sciences) using the manufacturer’s protocol.

### Forskolin treatment

HepG2 cells were grown overnight in presence of IFN-γ (1200U/ml) and IFN-β (1000U/ml) and following morning treated with forskolin (20 μM) in glucose free media for 4 hours.

### PTT, GTT and ITT

For pyruvate tolerance test (PTT), overnight fasted mice were injected with 2g/kg pyruvate (Sigma) through the intraperitoneal (i.p.) route. For the glucose tolerance test (GTT), overnight fasted mice were injected with 2 g/kg of glucose (Sigma) through the intraperitoneal (i.p.) route. For insulin tolerance test (ITT) 6 hour fasted mice were i.p. injected with 1.5U/kg recombinant human insulin (Sigma). After injection, tail vein blood glucose levels were monitored at different time intervals using the AlphaTRAK 2 blood glucose monitor.

### Insulin signalling assay

For insulin signalling studies, animals were fasted overnight and then injected through the i.p. route with recombinant human insulin (Sigma) at 1.5U/kg. Animals were sacrificed 15 minutes after injection and tissues were harvested and snap frozen in dry ice.

### Reanalysis of metabolomics data from public repository

The metabolomics data from the treatment cohort study from the [47] and the TB progression cohort study I.e., the GC6-74 study was downloaded from the Metabolomics Workbench database (project ID: PR000824). The data matrices were processed in MetaboAnalyst4.0 [83]. and new matrices containing the Branch chain amino acid (BCAA) based signature were subjected to Principal Component analysis and the components best distinguishing 2 groups are selected [49].

### Statistics

All animal experiments were done in duplicate with between 4–5 mice in each group. *In vitro* cell experiments were carried out in duplicate with a minimum of 3 replicates of each condition per experiment. Data are presented as the mean ± SEM. A 2-tailed Student’s *t* test was used to compare continuous variables between 2 groups. A 1-way ANOVA followed by Tukey’s multiple-comparison test was used to compare multiple groups. Any variation from the above is stated in the legends of each figure. GraphPad Prism 9 software was used for statistical analyses. All *P* values and sample sizes are reported in the figures or figure legends.

## Supporting information

S1 Fig Determination of immune cell (CD45+) populations in the livers of Mtb infected mice. Mice were aerosol exposed to Mtb as Fig 1 and the livers processed to obtain single cell suspension that were stained for flow cytometric analysis. Cells were gated by size, viability and the expression of the hemopoietic marker CD45 and the frequency of lineage labelled daughter populations determined. (a) Myeloid cells (markers CD11b, CD11c, Ly6G and siglecF) in uninfected, 21 day, and 42 day infected mice. (b) Lymphoid cells (CD19, TCRβ, CD4, CD8 –lineage markers and CD44, CD69 activation markers) in the uninfected, 21 day, and 42 day infected mice. The differences between the mean frequencies were determined by Kruskal Wallis test (n = 5–8, * = 0.05, ** = 0.01, *** = 0.001, **** = 0.0001).
(TIF)

S2 Fig (a) Principal component analysis of RNAseq data using all the variables of matrix (red-uninfected, green-21 day infected., blue 42 day infected) indicates changes in transcriptome level due to infection. (b) Volcano plots showing differential gene expression in 21 day (21D) and 42 day (42D) infected mice relative to uninfected control (UN). (c) Tissue enrichment analysis of the of gene sets associated with the clusters derived from K-means cluster analysis of the RNAseq data indicate partitioning of genes. (d) Principal component analysis of proteomics data using all the variables in data matrix (red-uninfected, green-21 day infected, blue 42 day infected) indicates changes of protein due to infection. e) Volcano plots showing differential protein expression in 21 day (21D) and 42 day (42D) infected mice relative to uninfected control (UN). (f) Correlation between transcriptomics and proteomics data. (g, h) Gene set enrichment analysis (GSEA) plot indicating loss of hepatocyte molecular identity (negative NES value) at gene (g) and protein (h) level.
(TIF)

S3 Fig RT-PCR analysis gluconeogenic gene of mouse liver from male 12 weeks old mice used in transcriptomics, proteomics and western blot analysis as in Figs 1 and 3 infected for 21 days (21D) and 42 days (42D). (b) RT-PCR analysis gluconeogenic gene of mouse liver from female 6 weeks old mice. (c) Western blot analysis to assess phosphorylation level of CREB in the female 6 weeks old mice infected with Mtb for 42 days. (d) Random and fasting tail vein glucose level in mouse infected with Mtb for 42 days (42D, n = 9) and uninfected controls (UN, n = 10). For statistical analysis Anova or Student *t*-test was used, *<0.05, **<0.01, ***<0.001.
(TIF)

S4 Fig Interferons secreted from immune cells activate Stat-1 signalling pathways in hepatocytes and upregulate interferon signalling gene expression such as Irf1. Interferon treatment also supresses phosphorylation of cAMP Response Element-Binding Protein (CREB) in hepatocytes which in turn reduces expression of gluconeogenic genes G6pc and Pck1.
(TIF)

S1 Table Percent weight change in mice used to generate the data in Fig 1. Uninfected mice and mice infected with *Mycobacterium tuberculosis* were held within the same isolators and exposed to the same environmental conditions and weighed to determine general health.
(DOCX)

S2 Table List of glucose/carbohydrate metabolic pathways enriched in cluster F&G of the transcriptomics data (Fig 1e) and cluster 4 of the proteomics data (bottom row) (Fig 1f). Details of the pathways and genes/proteins in the cluster can be found in Sheets F and G in both S2 and S3 Datas.
(DOCX)

S1 Data Details of the published human studies (n = 15) used in Fig 1a, which is a secondary analysis of changes in lipid and liver enzyme profiles of adult drug naïve TB patients relative to healthy controls. The file contains the study reference, blood parameter data derived from the studies and number of subjects in each group.
(XLSX)

S2 Data Details of the transcriptomics analysis. Sheets: a) Processed data matrix containing quantified information of all the genes used in transcriptomics analysis. b) List of genes in each cluster (Fig 1e) and their k-means score. List of genes representing cluster-A (c), cluster-B (d), cluster-C (e), cluster-D&E (f), cluster-F&G (g), cluster-H (h) and cluster-I&J (i).
(XLSX)

S3 Data Details of the transcriptomics analysis. Sheets: a) Processed data matrix containing quantified information of all the proteins (peptide IDs) used in transcriptomics analysis. b) List of proteins/peptide Ids in each cluster (Fig 1f) and their z-score. List of protein/peptide ids representing cluster-1 (c), cluster-2 (d), cluster-3 (e), cluster-4 (f) and cluster-5 (g). For better visualisation and clarity in the Fig 1f, the cluster names generated from Perseus analysis were renamed as cluster-1193 & cluster-310 to Cluster-1, cluster-1195 & cluster-310 to Cluster-2, cluster-1196 & cluster-1200 to Cluster-3, cluster-1197 & cluster-1156 to Cluster-4 and cluster-1199 to Cluster-5.
(XLSX)

S4 Data Details of genes used in ligand receptor analysis. For the column-D to column-I, number-1 represents presence of the ligand or receptor in the tissue and ‘0’ represents absence. From column-J to column-R, number-2 represents interaction between ligand and receptor, number-1 represents presence of either ligand or receptor without any interaction and ‘0’ represents both ligand and receptor absent.
(XLSX)
