# Supplementary figures and images for: Altered hepatic metabolic landscape and insulin sensitivity in response to pulmonary tuberculosis
Source: PLoS Pathog. 2024 Sep 27;20(9):e1012565. doi: 10.1371/journal.ppat.1012565 (PMC11463835; doi:10.1371/journal.ppat.1012565)

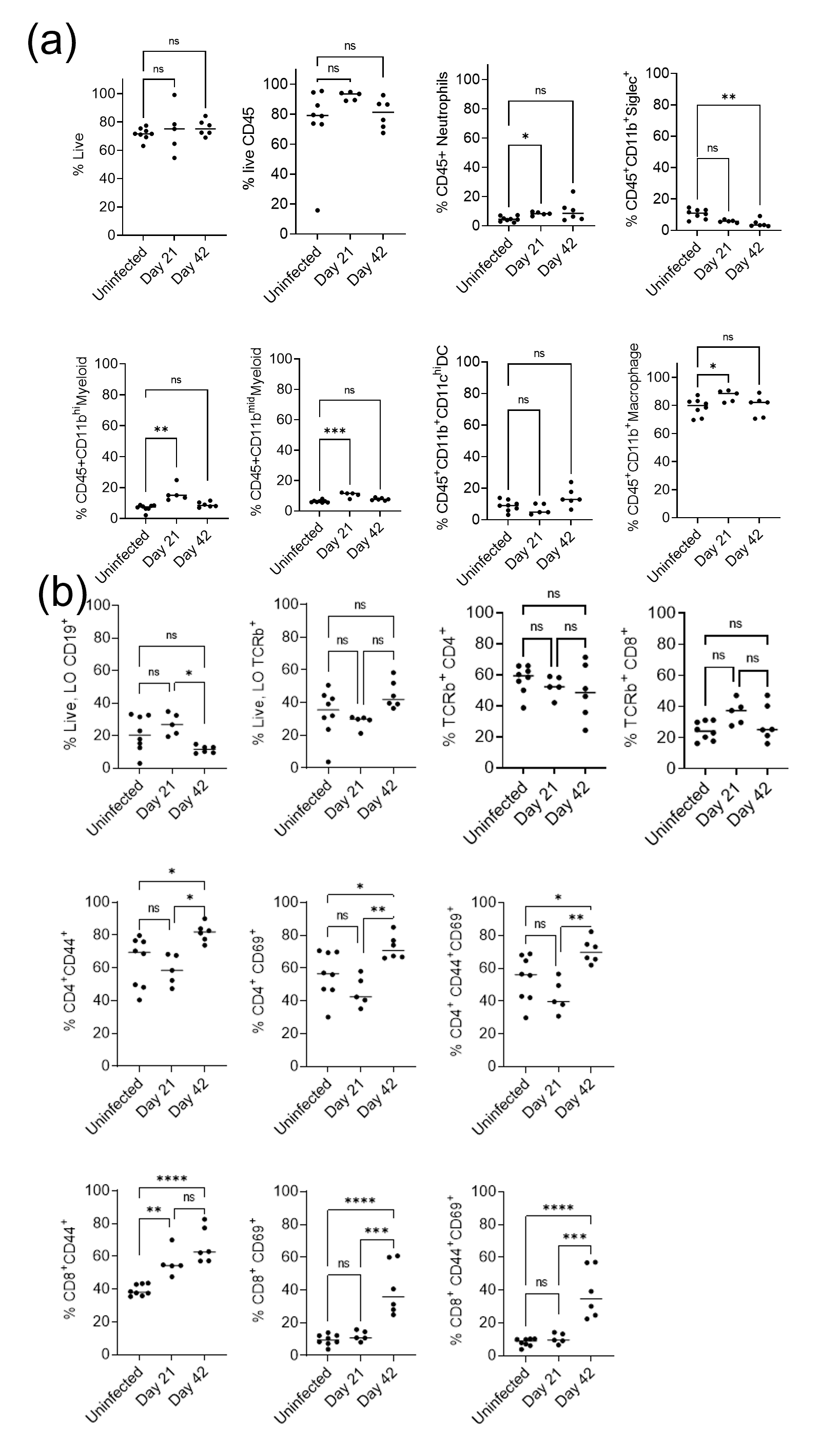

Supplement: S1 Fig — Mice were aerosol exposed to Mtb as Fig 1 and the livers processed to obtain single cell suspension that were stained for flow cytometric analysis. Cells were gated by size, viability and the expression of the hemopoietic marker CD45 and the frequency of lineage labelled daughter populations determined. (a) Myeloid cells (markers CD11b, CD11c, Ly6G and siglecF) in uninfected, 21 day, and 42 day infected mice. (b) Lymphoid cells (CD19, TCRβ, CD4, CD8 –lineage markers and CD44, CD69 activation markers) in the uninfected, 21 day, and 42 day infected mice. The differences between the mean frequencies were determined by Kruskal Wallis test (n = 5–8, * = 0.05, ** = 0.01, *** = 0.001, **** = 0.0001). (TIF) [file ppat.1012565.s001.tif]

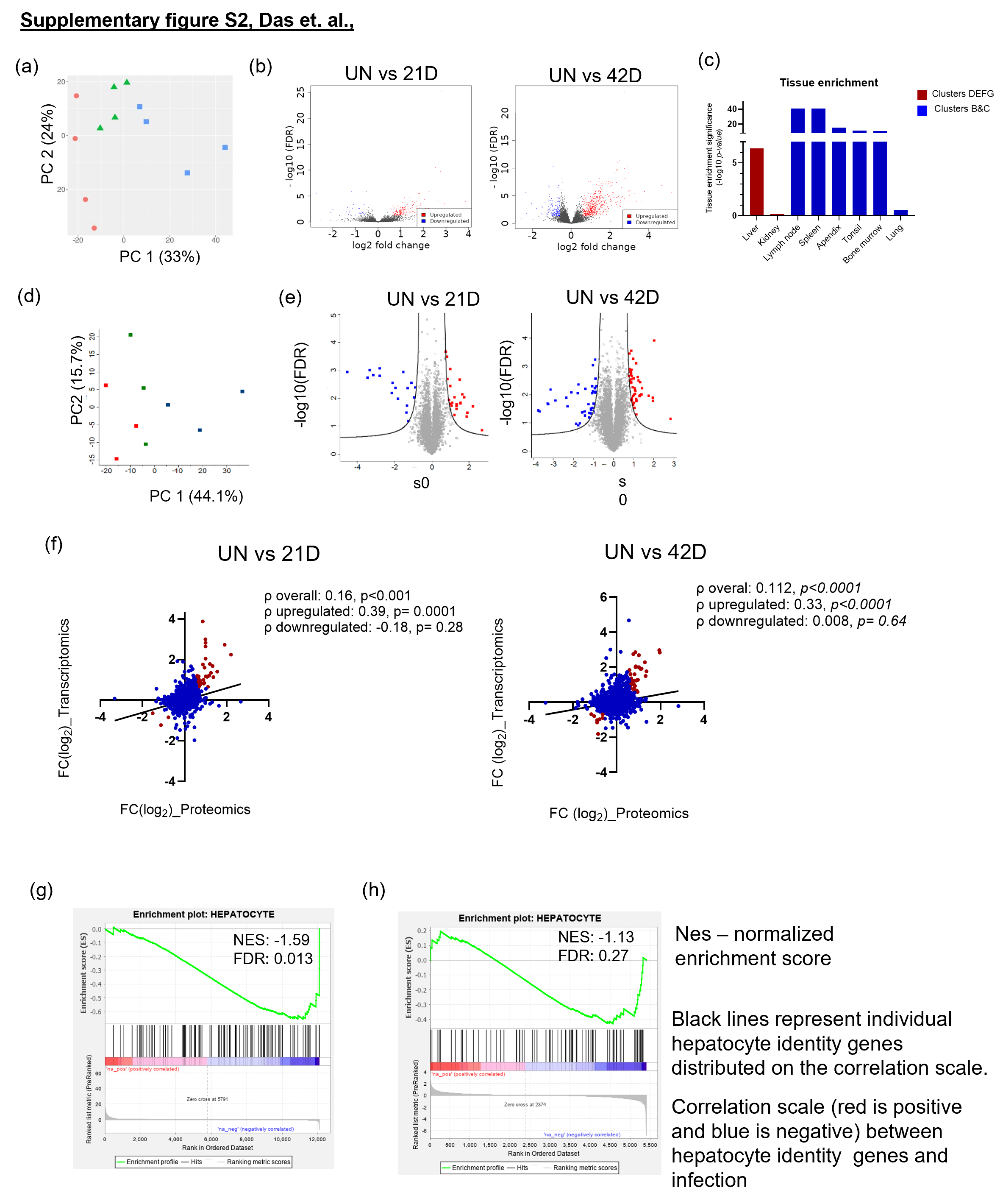

Supplement: S2 Fig — (a) Principal component analysis of RNAseq data using all the variables of matrix (red-uninfected, green-21 day infected., blue 42 day infected) indicates changes in transcriptome level due to infection. (b) Volcano plots showing differential gene expression in 21 day (21D) and 42 day (42D) infected mice relative to uninfected control (UN). (c) Tissue enrichment analysis of the of gene sets associated with the clusters derived from K-means cluster analysis of the RNAseq data indicate partitioning of genes. (d) Principal component analysis of proteomics data using all the variables in data matrix (red-uninfected, green-21 day infected, blue 42 day infected) indicates changes of protein due to infection. e) Volcano plots showing differential protein expression in 21 day (21D) and 42 day (42D) infected mice relative to uninfected control (UN). (f) Correlation between transcriptomics and proteomics data. (g, h) Gene set enrichment analysis (GSEA) plot indicating loss of hepatocyte molecular identity (negative NES value) at gene (g) and protein (h) level. (TIF) [file ppat.1012565.s002.tif]

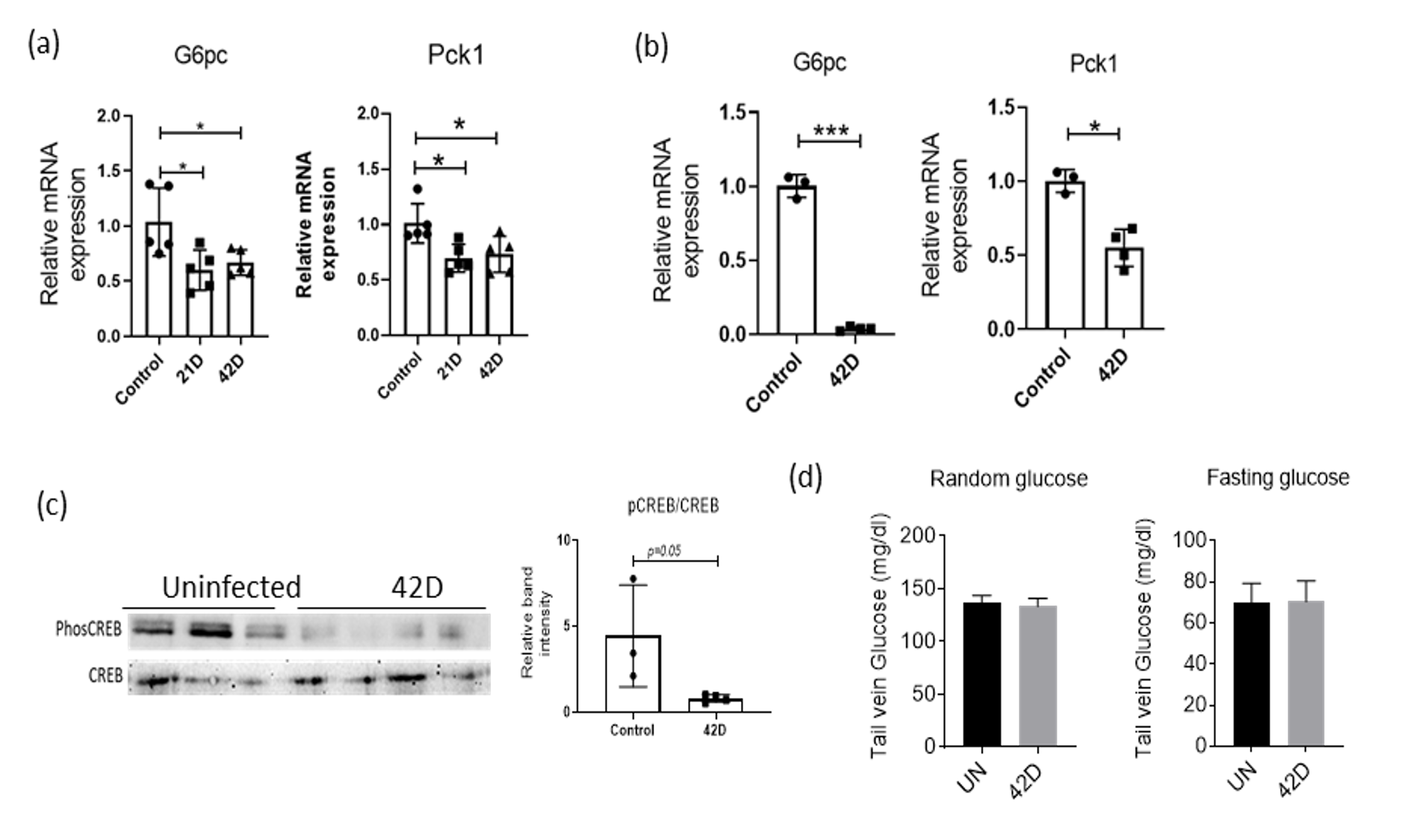

Supplement: S3 Fig — RT-PCR analysis gluconeogenic gene of mouse liver from male 12 weeks old mice used in transcriptomics, proteomics and western blot analysis as in Figs 1 and 3 infected for 21 days (21D) and 42 days (42D). (b) RT-PCR analysis gluconeogenic gene of mouse liver from female 6 weeks old mice. (c) Western blot analysis to assess phosphorylation level of CREB in the female 6 weeks old mice infected with Mtb for 42 days. (d) Random and fasting tail vein glucose level in mouse infected with Mtb for 42 days (42D, n = 9) and uninfected controls (UN, n = 10). For statistical analysis Anova or Student t-test was used, *<0.05, **<0.01, ***<0.001. (TIF) [file ppat.1012565.s003.tif]

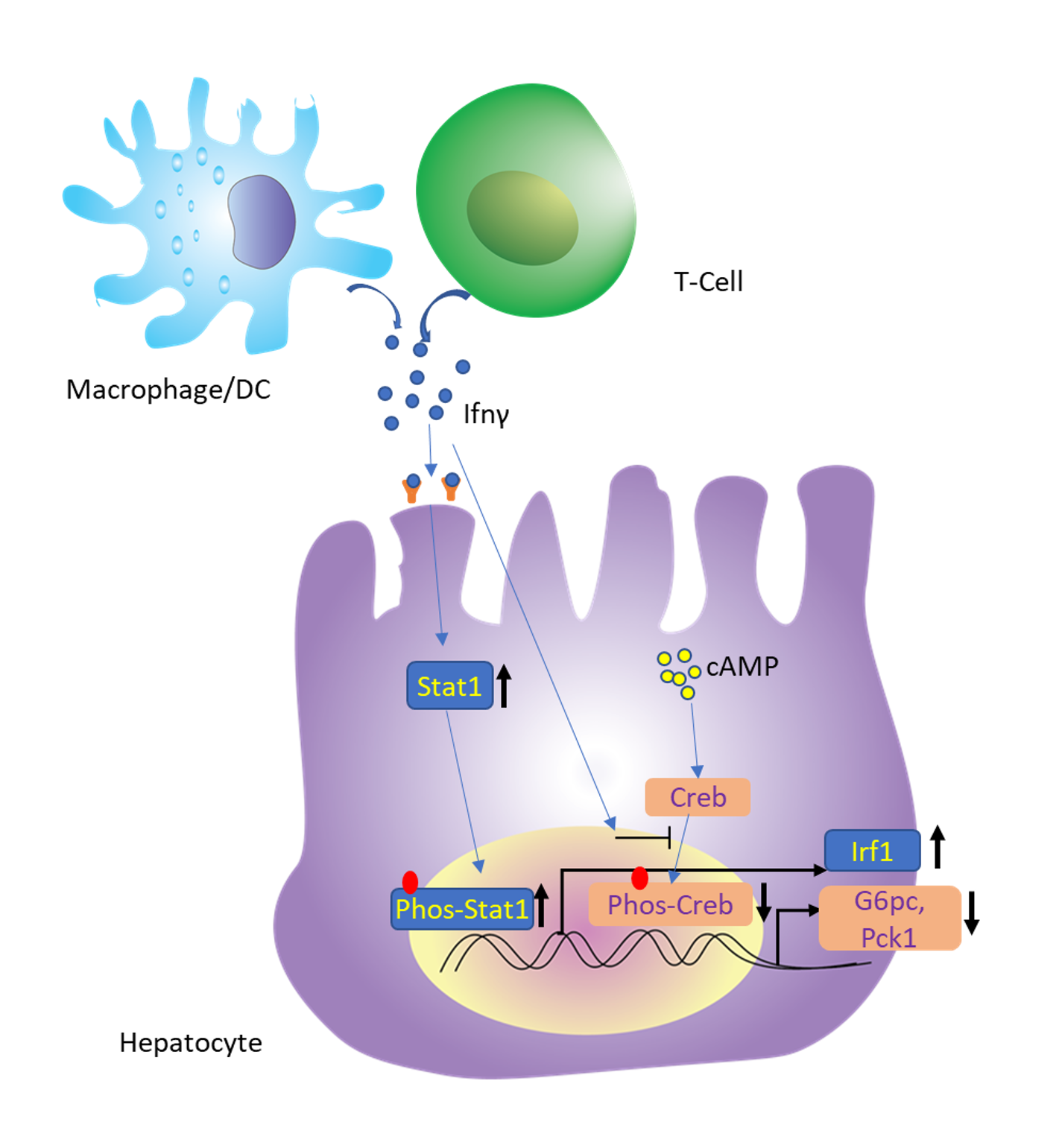

Supplement: S4 Fig — Interferon treatment also supresses phosphorylation of cAMP Response Element-Binding Protein (CREB) in hepatocytes which in turn reduces expression of gluconeogenic genes G6pc and Pck1. (TIF) [file ppat.1012565.s004.tif]
