# Supplementary material for: Altered hepatic metabolic landscape and insulin sensitivity in response to pulmonary tuberculosis
Source: PLoS Pathog. 2024 Sep 27;20(9):e1012565. doi: 10.1371/journal.ppat.1012565 (PMC11463835; doi:10.1371/journal.ppat.1012565)
Supplement: S1 Table — Uninfected mice and mice infected with Mycobacterium tuberculosis were held within the same isolators and exposed to the same environmental conditions and weighed to determine general health. (DOCX) [file ppat.1012565.s005.docx]

S1 Table. Percent weight change in mice used to generate the data in Figure 1. Uninfected mice and mice infected with *Mycobacterium tuberculosis* were held within the same isolators and exposed to the same environmental conditions and weighed to determine general health.

|  | Percent weight change from day 6 | | Significance |
| --- | --- | --- | --- |
| Day post infection | Uninfected (n=10) | Infected (n=10-20) | *P* value (Mann Whitney) |
| 6 | 100 | 100 |  |
| 15 | 103 | 103 | 0.8291 |
| 21 | 102 | 103 | 0.3619 |
| 28 | 105 | 103 | 0.1962 |
| 39 | 105 | 104 | 0.5365 |
