# Supplementary material for: Altered hepatic metabolic landscape and insulin sensitivity in response to pulmonary tuberculosis
Source: PLoS Pathog. 2024 Sep 27;20(9):e1012565. doi: 10.1371/journal.ppat.1012565 (PMC11463835; doi:10.1371/journal.ppat.1012565)
Supplement: S2 Table — Details of the pathways and genes/proteins in the cluster can be found in Sheets F and G in both S2 and S3 Datas. (DOCX) [file ppat.1012565.s006.docx]

S2 Table. List of glucose/carbohydrate metabolic pathways enriched in cluster F&G of the transcriptomics data (Fig 1e) and cluster 4 of the proteomics data (bottom row) (Fig 1f). Details of the pathways and genes/proteins in the cluster can be found in Sheets F and G in both S2 and S3 Data.

| **source** | **Pathway name** | **term_id** | **adj *p* value** | **neg log10**  **of adj *p* value** | **Size of pathway** | **query**  **size** | **input genes/pathway** |
| --- | --- | --- | --- | --- | --- | --- | --- |
| GO:BP | regulation of glucose metabolic process | GO:0010906 | 3.94E-05 | 4.403979 | 114 | 405 | 13 |
| GO:BP | regulation of carbohydrate metabolic process | GO:0006109 | 0.000161 | 3.793059 | 201 | 405 | 16 |
| GO:BP | glucose metabolic process | GO:0006006 | 0.000669 | 3.174675 | 223 | 405 | 16 |
| GO:BP | gluconeogenesis | GO:0006094 | 0.007137 | 2.146507 | 102 | 405 | 10 |
| GO:BP | regulation of gluconeogenesis | GO:0006111 | 0.011219 | 1.950037 | 64 | 405 | 8 |
| GO:BP | carbohydrate metabolic process | GO:0005975 | 1.38E-13 | 12.86099 | 599 | 184 | 29 |
